# Supplementary material for: Moringa oleifera Lam. Isothiocyanate Quinazolinone Derivatives Inhibit U251 Glioma Cell Proliferation through Cell Cycle Regulation and Apoptosis Induction
Source: Int J Mol Sci. 2023 Jul 12;24(14):11376. doi: 10.3390/ijms241411376 (PMC10379366; doi:10.3390/ijms241411376)
Supplement: Supplementary file 1 [file ijms-24-11376-s001.zip › supplementary Materials S1.pdf]

Supplementary Material S1:

Supplementary Figure S1

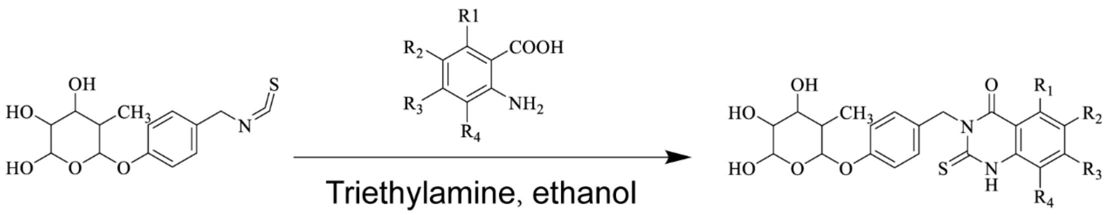

Figure S1. 4-α-L-rhamnosyl-benzyl isothiocyanate reacted with o-aminobenzoic acid

Supplementary Figure S2

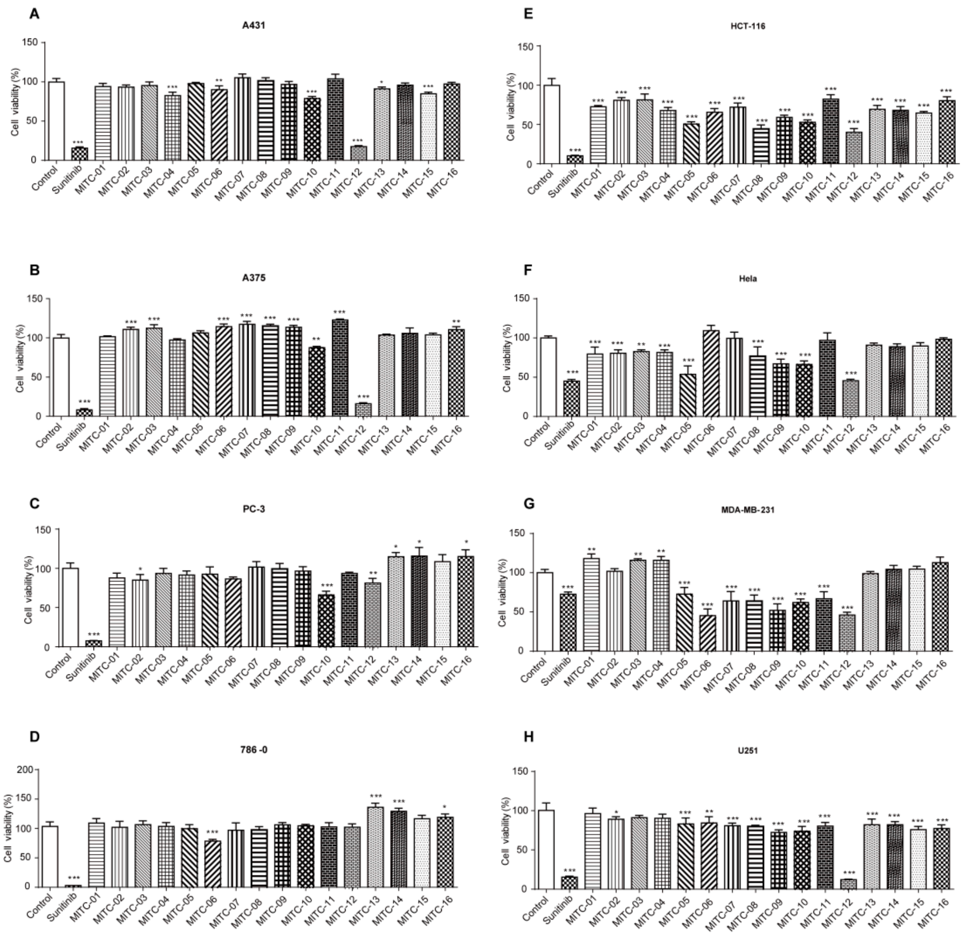

Figure S2. The viability of 8 types of cancer cells after treatment with 16 types of MITC quinazolinone derivatives (10 μM) for 48 h. A-G: A431, A375, PC-3, 786-O, HCT-116, Hela, MDA-MB-231 and U251 cells (Sunitinib as positive control). Data are expressed as mean ± SEM. * indicates significant difference compared to control group, **p* < 0.05, ***p* < 0.01, ****p* < 0.001.

Supplementary Figure S3

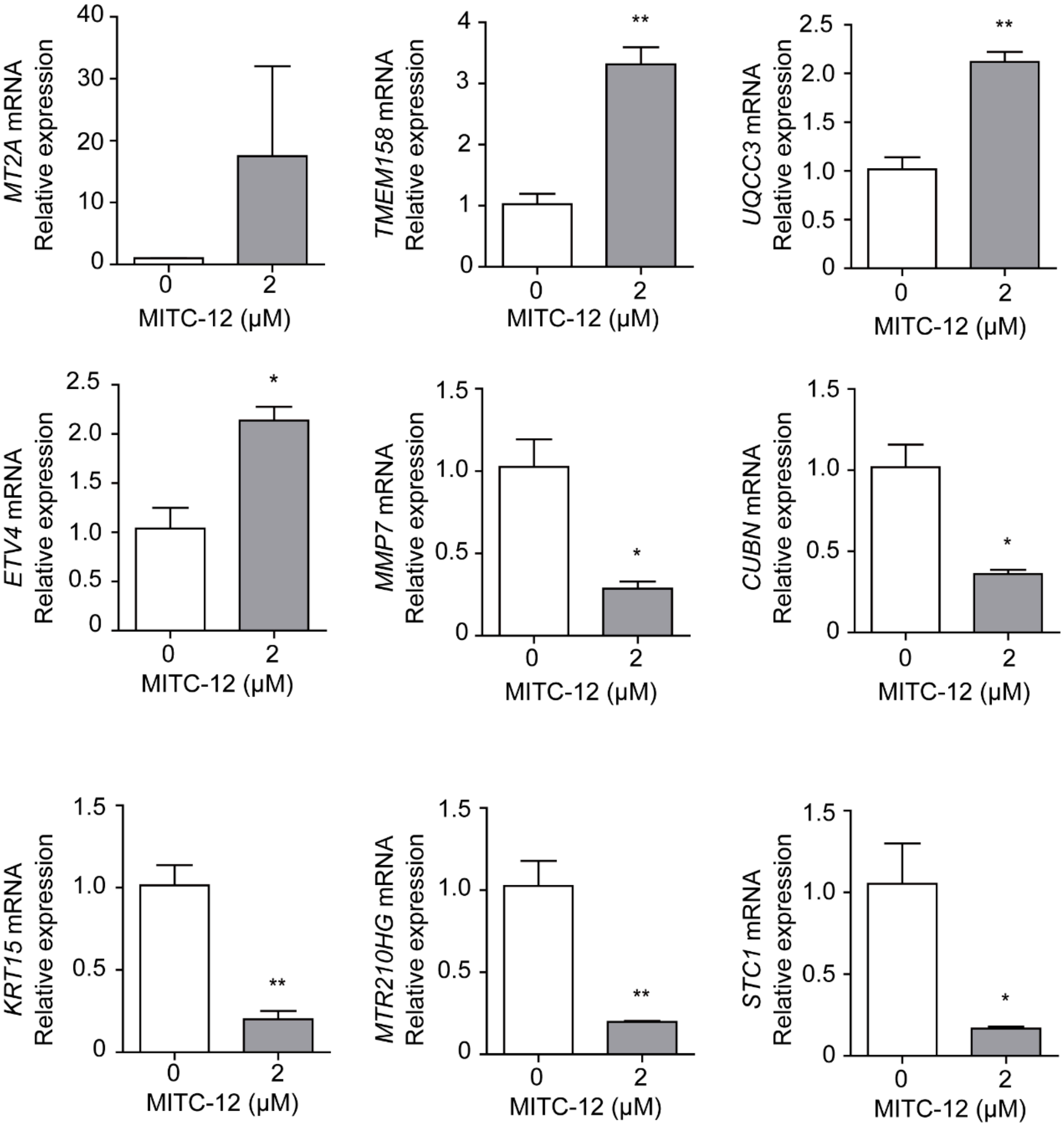

Figure S3. Validation of differentially expressed genes by RT-qPCR. U251 cells were treated with MITC-12 (0, 2 μM) for 24 h，effect of MTTC-12 on mRNA expression of *MT2A*, *TMEM158*, *UQCC3*, *ETV4*, *MMP7*, *CUBN*, *KRT15*, *MIR210HG*, *STC1*. Data are expressed as mean ± SEM. * indicates significant difference compared to control group, *$p < 0.05$, **$p < 0.01$.

Supplementary Table S1:

Table S1. Structural formula of sixteen MITC quinazolinone derivatives

MITC number	Substituents and Substitutes			
	R_1	R_2	R_3	R_4
MITC-01	H	H	NO_2	H
MITC-02	H	F	H	H
MITC-03	H	H	OCH_3	H
MITC-04	H	H	Br	H
MITC-05	H	H	Cl	H
MITC-06	H	Cl	H	H
MITC-07	H	H	F	H
MITC-08	H	H	H	Cl
MITC-09	H	CH_3	H	Br
MITC-10	H	I	H	H
MITC-11	F	H	H	H
MITC-12	H	Br	H	Br
MITC-13	H	H	H	F
MITC-14	H	H	H	OCH_3
MITC-15	H	OCH_3	OCH_3	H
MITC-16	H	OH	H	H

Supplementary Table S2：

Table S2. Properties of sixteen MITC quinazolinone derivatives

MITC number	Reaction temperature (°C)	Products				
		Color and shape	Productivity (%)	Purity (%)	Molecular weight	Chemical formula
MITC-01	90	Gray-brown powder	70.4	99.37	475.10	$C_{21}H_{21}N_3O_8S$
MITC-02	90	Gray-brown powder	67.6	96	448.11	$C_{21}H_{21}FN_2O_6S$
MITC-03	90	Brown powder	19.4	93	460.13	$C_{22}H_{24}N_2O_7S$
MITC-04	90	Brown powder	52.8	91	508.03	$C_{21}H_{21}BrN_2O_6S$
MITC-05	90	Gray-brown powder	21.2	100	446.08	$C_{21}H_{21}ClN_2O_6S$
MITC-06	90	Gray-brown powder	29.2	85	464.08	$C_{21}H_{21}ClN_2O_6S$
MITC-07	90	Grey powder	24.8	85	448.11	$C_{21}H_{21}FN_2O_6S$
MITC-08	90	Gray-brown powder	46.4	98	446.08	$C_{21}H_{21}ClN_2O_6S$
MITC-09	90	Gray-brown powder	72.4	95	522.50	$C_{22}H_{23}BrN_3O_6S$
MITC-10	90	Grey -white powder	74.6	95	556.02	$C_{21}H_{21}IN_2O_6S$
MITC-11	90	Brown powder	56.8	97	448.11	$C_{21}H_{21}FN_2O_6S$
MITC-12	90	Grey powder	20.4	91	585.94	$C_{21}H_{20}Br_2N_2O_6S$
MITC-13	90	Gray-brown powder	19.6	94	448.11	$C_{21}H_{21}FN_2O_6S$
MITC-14	90	Gray-brown powder	68.4	97	460.13	$C_{22}H_{24}N_2O_7S$
MITC-15	90	Grey powder	87.0	96	490.14	$C_{23}H_{24}N_2O_8S$
MITC-16	90	Brown powder	61.8	100	446.11	$C_{21}H_{22}N_2O_7S$

Supplementary Table S3：

Table S3. Primer sequence table

Gene	Forward	Reverse
MT2A	GTGGGCTGTGCCAAGTGT	CAAACGGTCACGGTCAGG
TMEM158	GCTGCATTTCTGCTGCCTA	TCCACACCACGATGACCA
UQCC3	CCAAGGAGCAGGGAGGA	CGCCAACCATCCAGTTCT
ETV4	CCGATACTATTATGAGAAAGGC	CATCCAAGTGGGACAAAGG
MMP7	GGGAACAGGCTCAGGAC	TCTATGACGCGGGAGTTT
CUBN	CCGAACGTGTGCCTATG	GAGGTGAAGCGGATGAAC
KRT15	TTAGGGGAGGAGACGAGGT	CTGGAGCCCGTGAGTTCT
MIR210HG	CACACACGGCCTTTCTCA	GCATGGACTCGGACTGG
STC1	ATCAAACGCACATCCCA	AAAATCAAACCAGGCACAG
GAPDH	GTGGGCTACACTGAGCACC	AAGTGGTCGTTGAGGGCAATG
