# Supplementary material for: Moringa oleifera Lam. Isothiocyanate Quinazolinone Derivatives Inhibit U251 Glioma Cell Proliferation through Cell Cycle Regulation and Apoptosis Induction
Source: Int J Mol Sci. 2023 Jul 12;24(14):11376. doi: 10.3390/ijms241411376 (PMC10379366; doi:10.3390/ijms241411376)
Supplement: Supplementary file 1 [file ijms-24-11376-s001.zip › supplementary Materials S2.pdf]

Supplementary Material S2:

NMR result of sixteen MITC isothiocyanate derivatives

^{13}C NMR of MITC-01

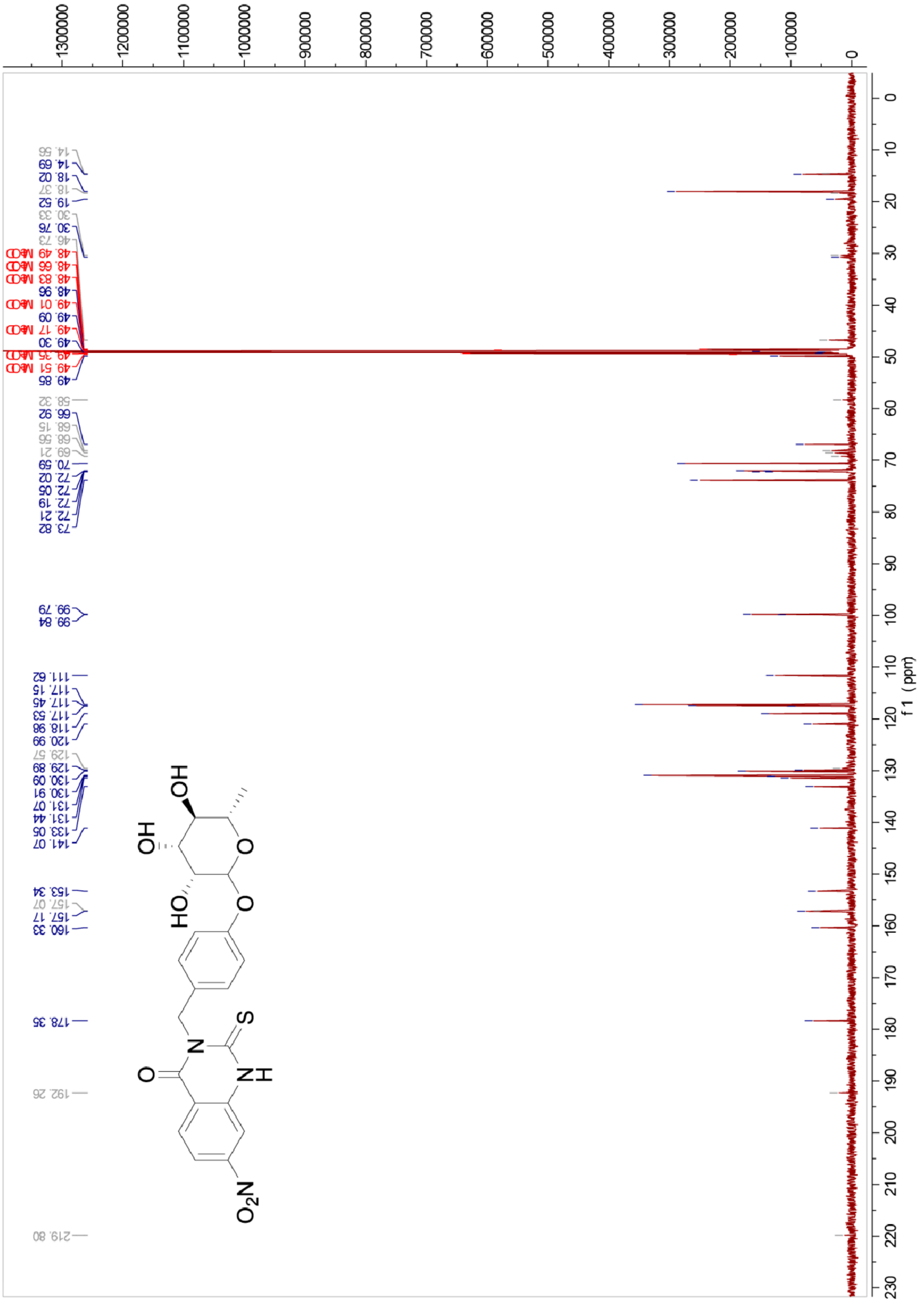

^{1}H NMR of MITC-01

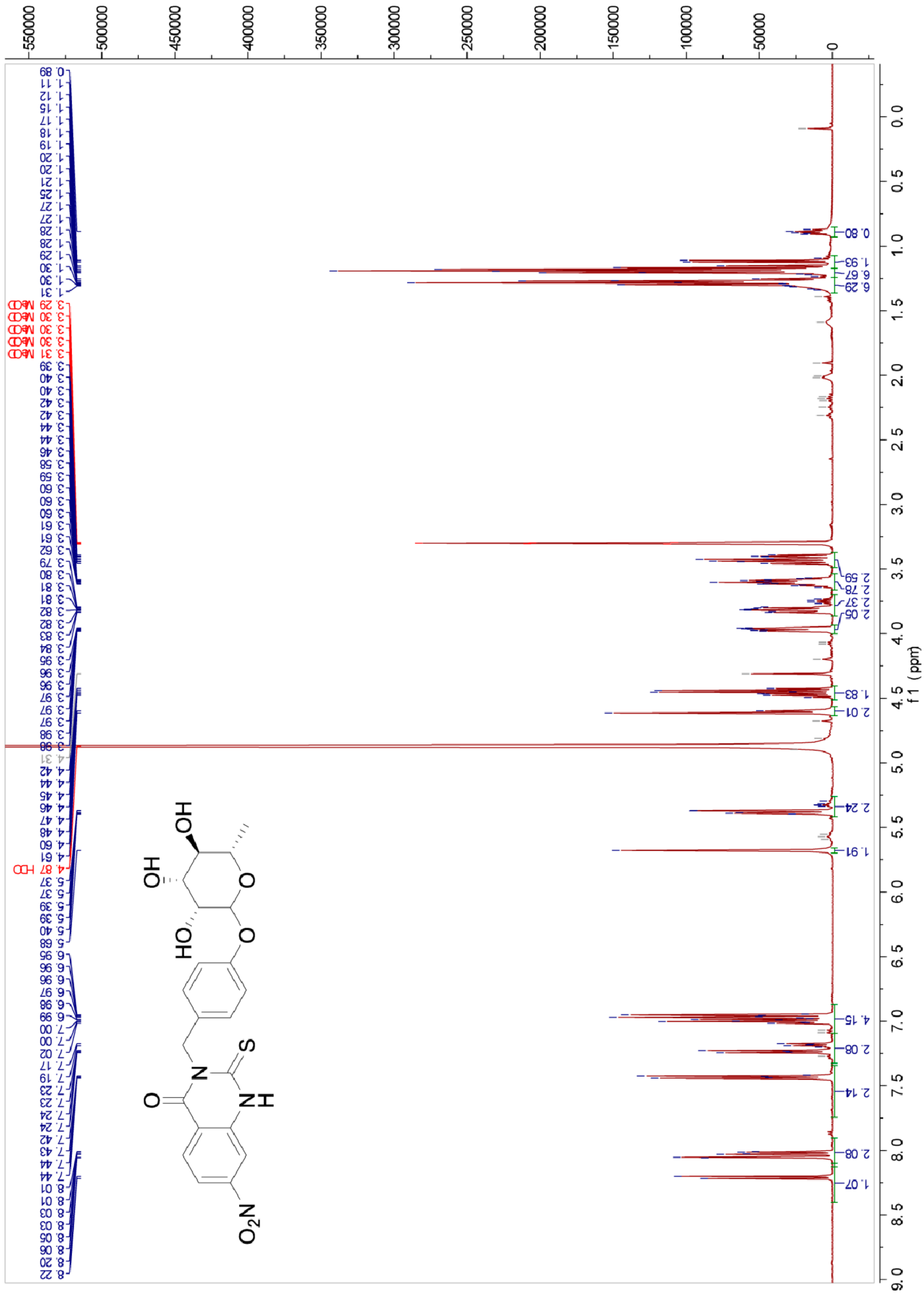

NMR result of MITC-01

^{1}H NMR (500 MHz, Mode) δ 8.21 (d, J = 8.6 Hz, 1H), 8.05 (d, J = 2.1 Hz, 1H), 8.02 (dd, J = 8.6, 2.1 Hz, 1H), 7.45 – 7.41 (m, 2H), 7.25 – 7.22 (m, 1H), 6.97 – 6.94 (m, 2H), 5.68 (s, 2H), 5.39 (d, J = 2.1 Hz, 1H), 5.37 (d, J = 2.1 Hz, 1H), 4.61 (s, 1H), 4.45 (d, J = 7.1. Hz, 1H), 3.96 (1H), 3.81 1H), 3.61 – 3.58 (m, 1H), 3.42 – 3.37 (m, 1H), 1.28 (m, J = 7.1, 1.4 Hz, 3H).; ^{13}C NMR (126 MHz, MeOD) δ 192.3, 178.3, 160.3, 157.1, 153.3, 141.1, 133.1, 131.4, 131.1, 129.8, 120.9, 118.9, 117.5, 111.6, 99.7, 73.8, 72.2, 72.1, 70.5, 48.4, 18.1.

^{13}C NMR of MITC-02

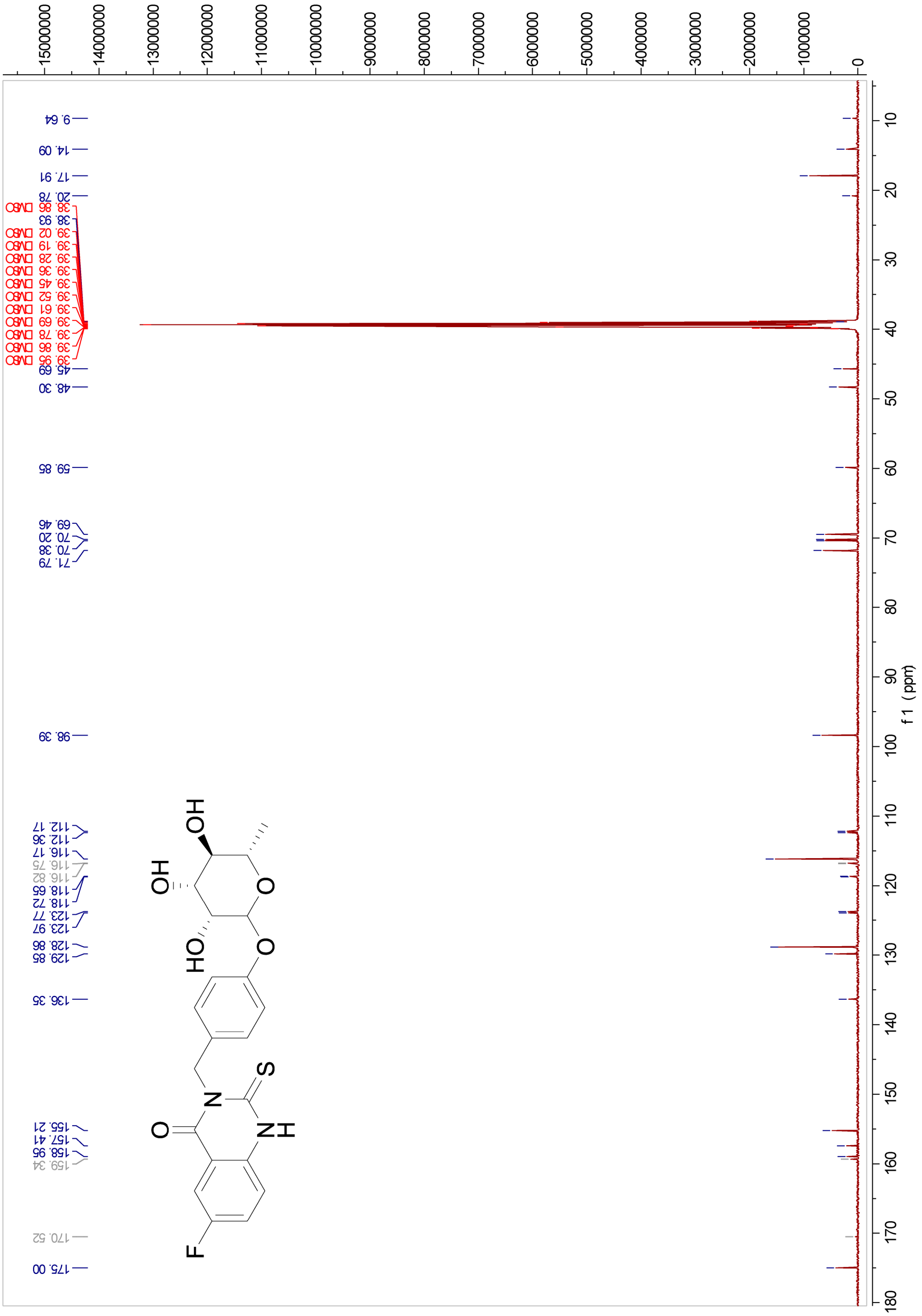

^{1}H NMR of MITC-02

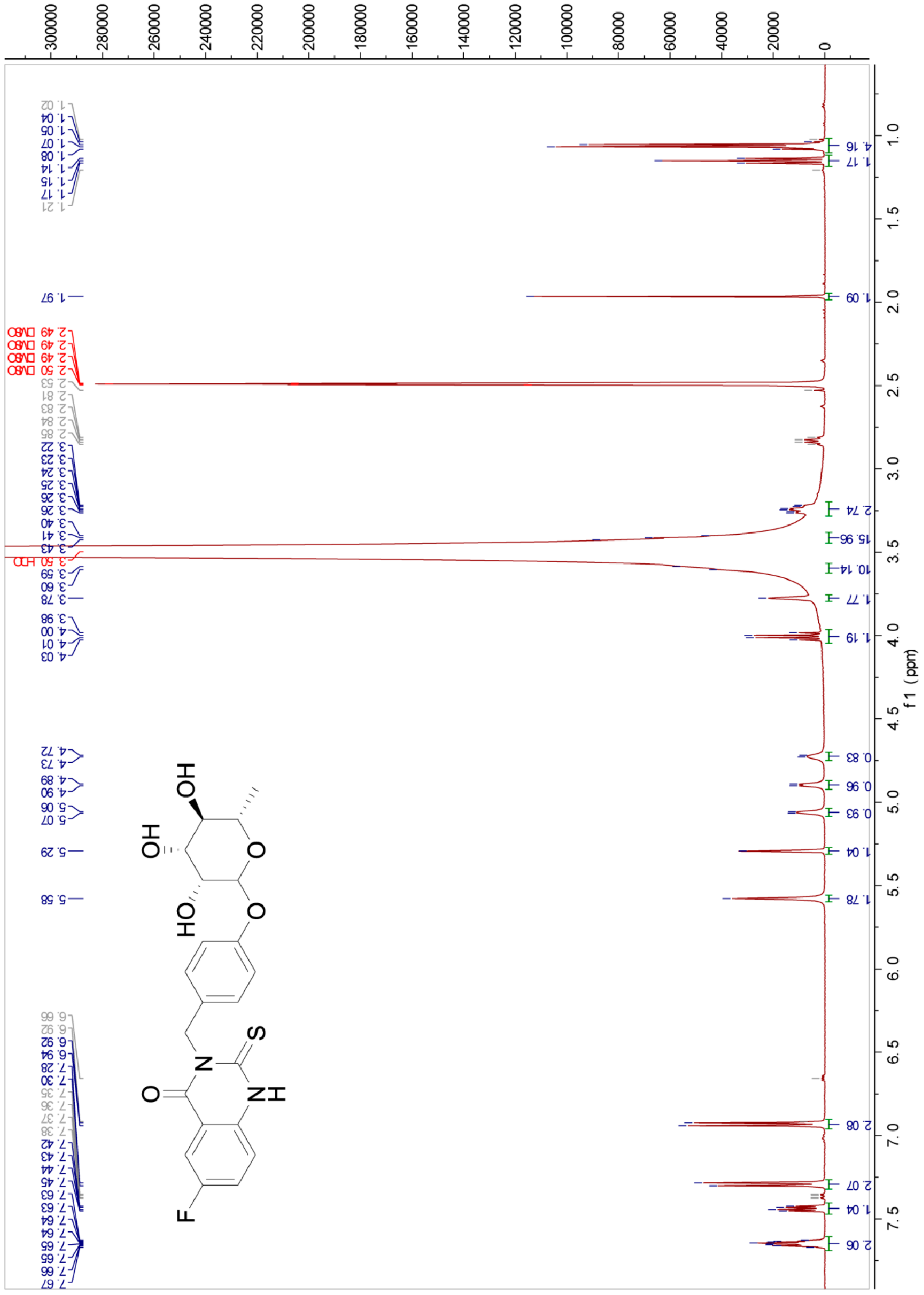

NMR result of MITC-02

^{1}H NMR (500 MHz, DMSO-d_6) δ 7.65 (dtt, J = 8.6, 5.6, 3.0 Hz, 2H), 7.44 (dd, J = 9.8, 4.5 Hz, 1H), 7.29 (d, J = 8.8 Hz, 2H), 6.96 – 6.90 (m, 2H), 5.58 (s, 2H), 5.29 (d, J = 1.6 Hz, 1H), 5.06 (s, 1H), 4.90 (d, J = 5.2 Hz, 1H), 4.72 (s, 1H), 4.00 (1H), 3.78 (1H), 3.24 (1H), 1.05 (m, J = 7.6 Hz, 3H)..; ^{13}C NMR (126 MHz, DMSO) δ 175.0, 159.3, 158.9, 157.4, 155.2, 136.3, 129.8, 128.8, 123.9, 116.8, 116.1, 112.1, 98.3, 71.7, 70.3, 69.4, 59.8, 48.3, 17.9.

^{13}C NMR of MITC-03

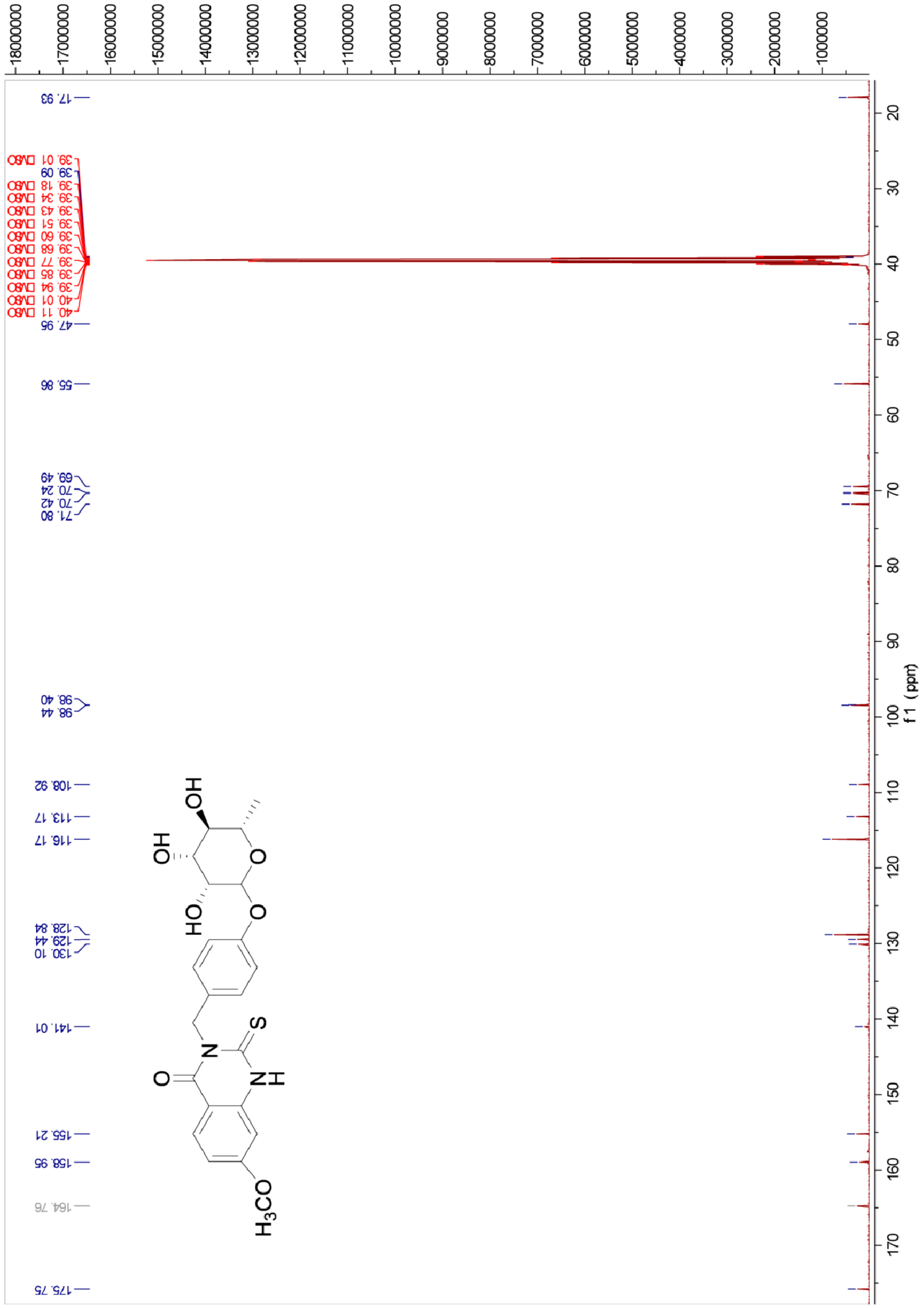

^{1}H NMR of MITC-03

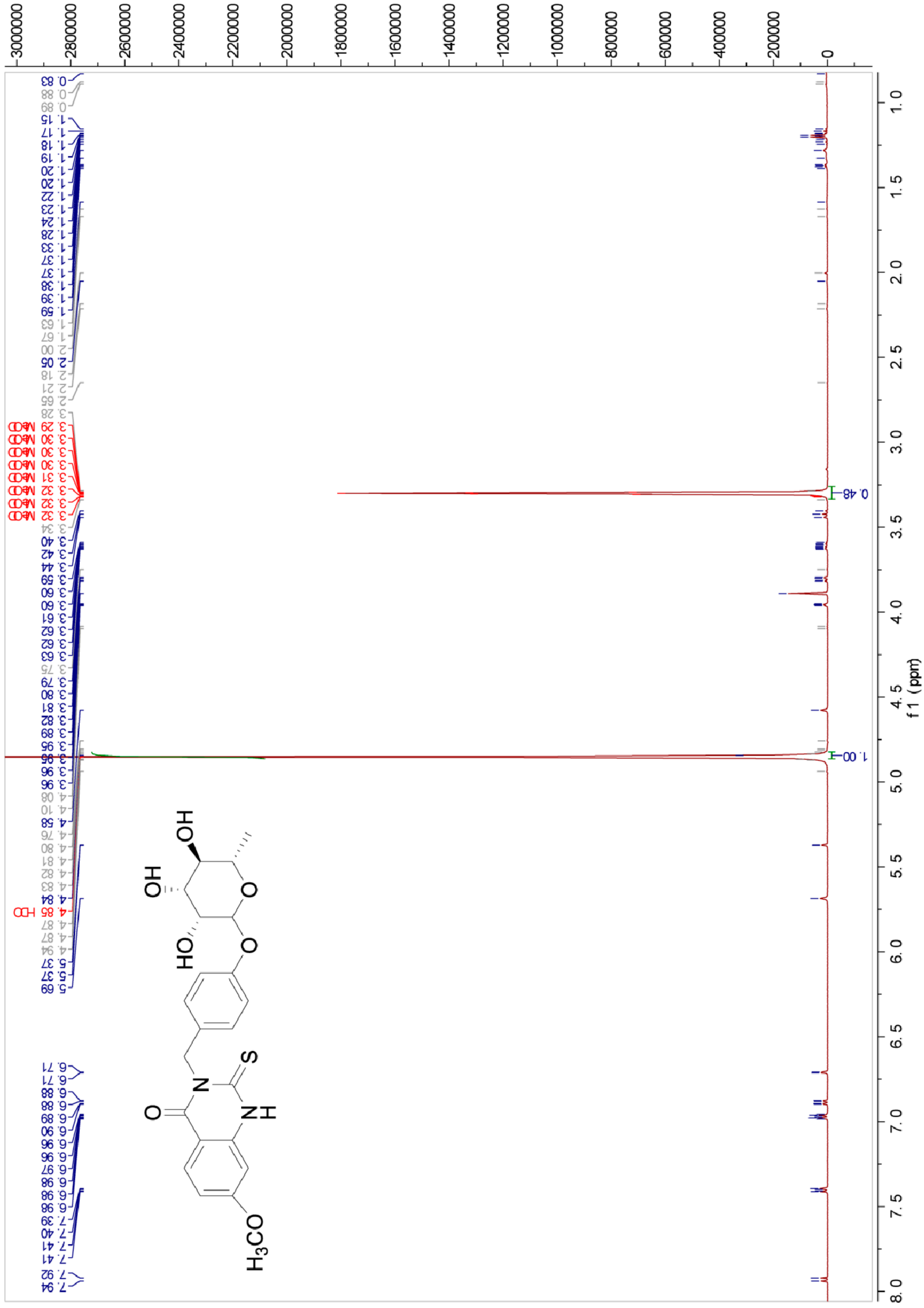

NMR result of MITC-03

^{1}H NMR (500 MHz, DMSO) δ 7.93 (d, J = 8.9 Hz, 1H), 7.43 – 7.37 (m, 2H), 7.01 – 6.95 (m, 2H), 6.89 (dd, J = 8.9, 2.4 Hz, 1H), 6.71 (d, J = 2.4 Hz, 1H), 5.69 (s, 2H), 5.37 (d, J = 1.8 Hz, 1H), 4.58 (s, 2H), 3.96 (dd, J = 3.5, 1.8 Hz, 1H), 3.89 (s, 3H), 3.81 (1H), 3.65 – 3.58 (m, 1H), 3.47 – 3.37 (m, 1H), 2.03 (1H), 1.20 (d, J = 6.3 Hz, 3H)..; ^{13}C NMR (126 MHz, DMSO) δ 175.7, 164.7, 158.9, 155.2, 140.9, 130.0, 129.4, 128.8, 120.7, 116.1, 113.1, 108.9, 98.4, 71.8, 70.4, 70.2, 69.4, 55.8, 47.9, 17.9.

^{13}C NMR of MITC-04

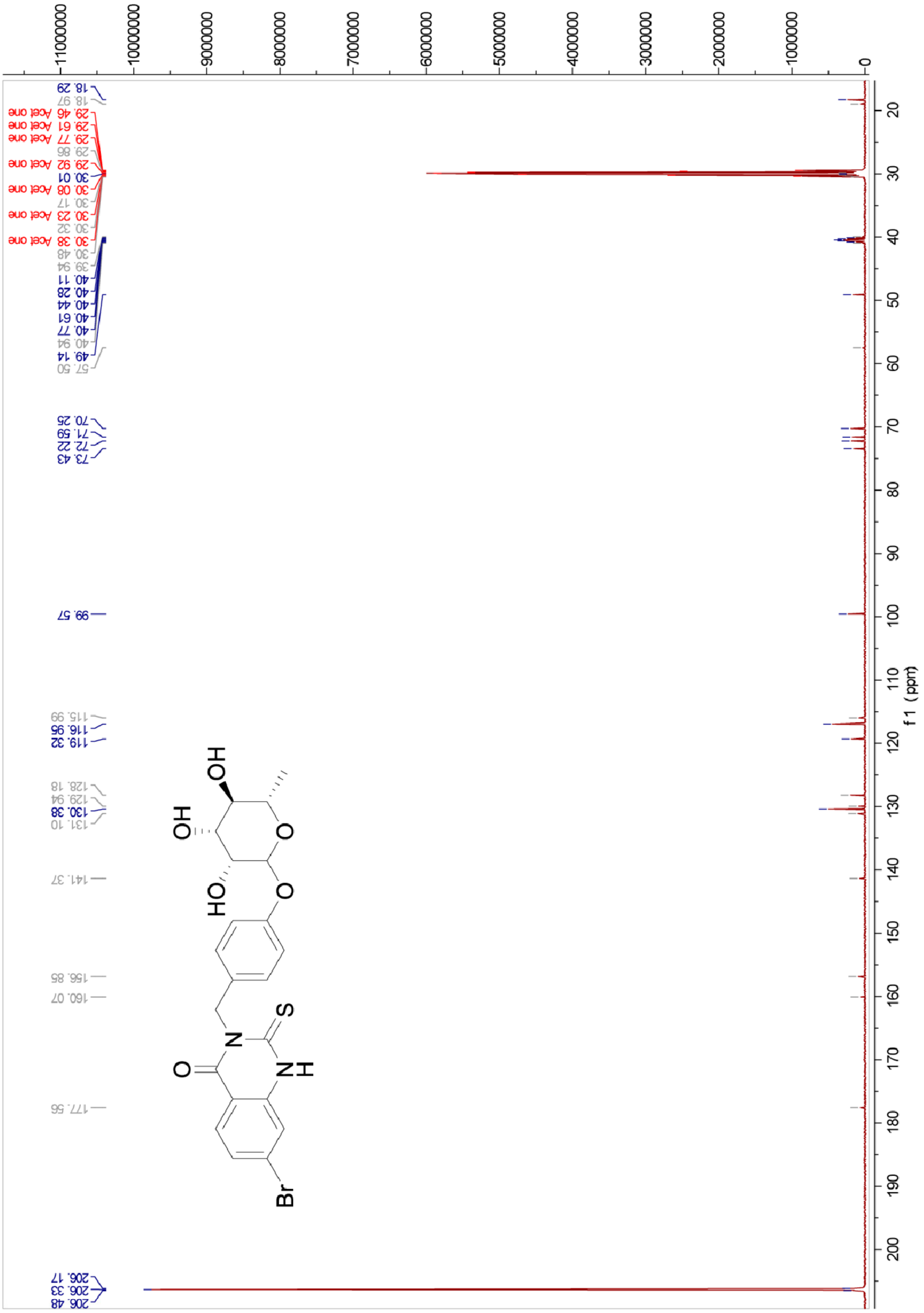

^{1}H NMR of MITC-04

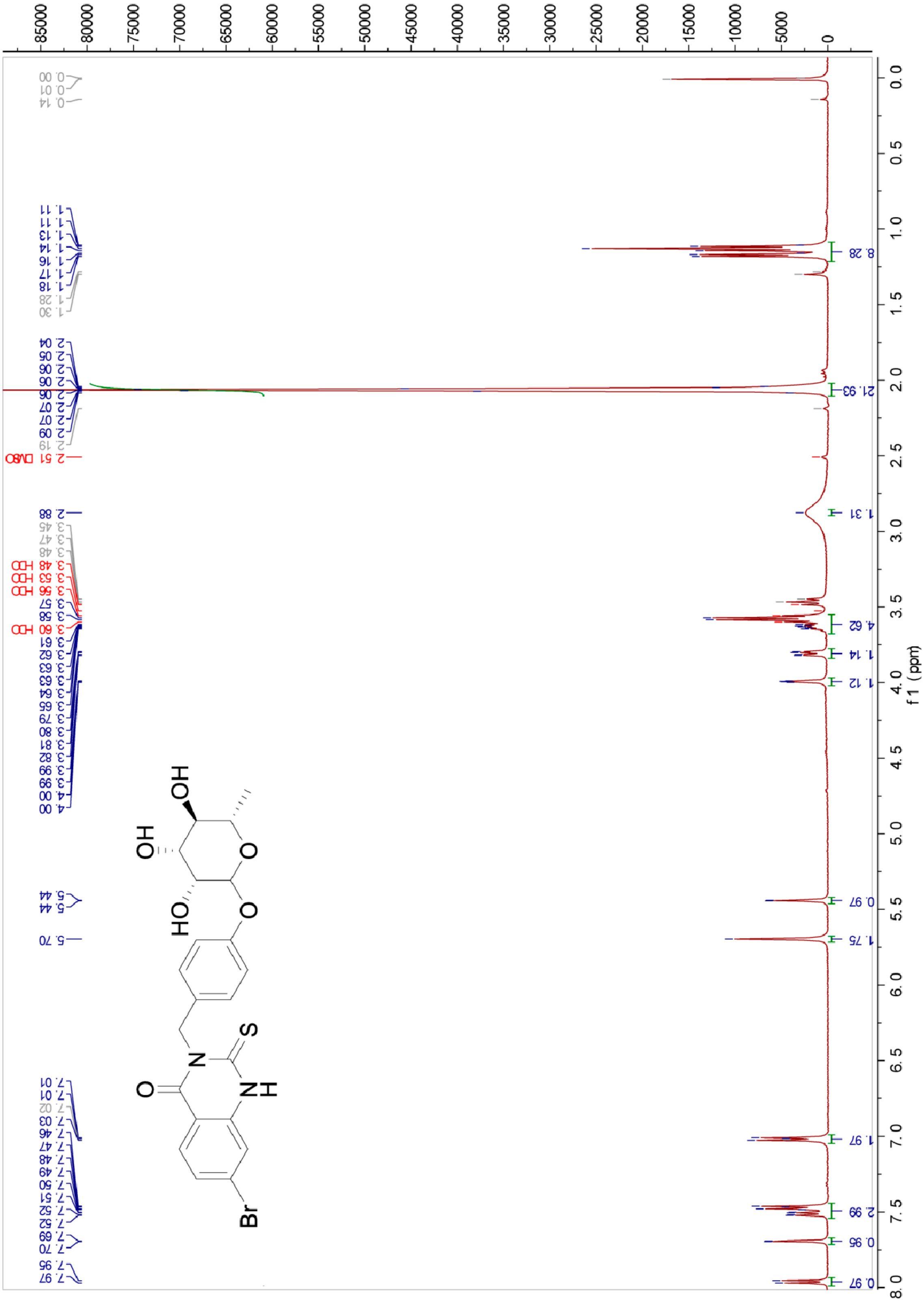

NMR result of MITC-04

^{1}H NMR (500 MHz, DMSO-d_6) δ 7.96 (d, J = 8.5 Hz, 1H), 7.69 (d, J = 1.4 Hz, 1H), 7.54 – 7.49 (m, 1H), 7.47 (d, J = 8.5 Hz, 2H), 7.02 (d, J = 8.6 Hz, 2H), 5.70 (s, 2H), 5.44 (s, 1H), 3.99 (s, 1H), 3.81 (dd, J = 9.2, 3.4 Hz, 1H), 3.63 (1H), 3.58 (s, 1H), 3.57 (s, 1H), 3.47 (1H), 1.18 (d, J = 6.2 Hz, 3H)..; ^{13}C NMR (126 MHz, Acetone) δ 177.5, 160.0, 156.8, 141.3, 131.1, 130.3, 129.9, 128.1, 119.3, 116.9, 115.9, 99.5, 73.4, 72.2, 71.5, 70.2, 49.1, 18.2.

^{13}C NMR of MITC-05

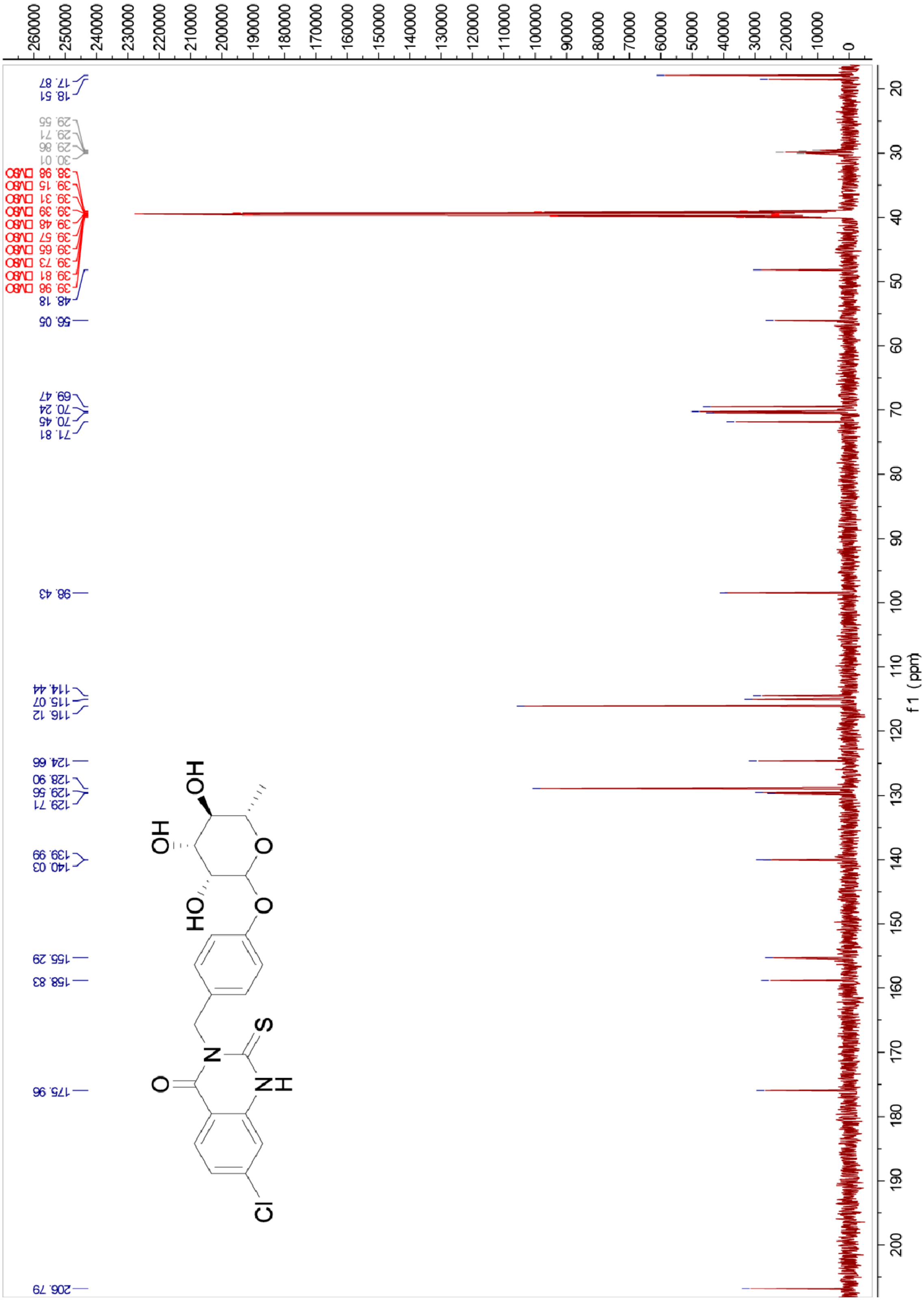

^{1}H NMR of MITC-05

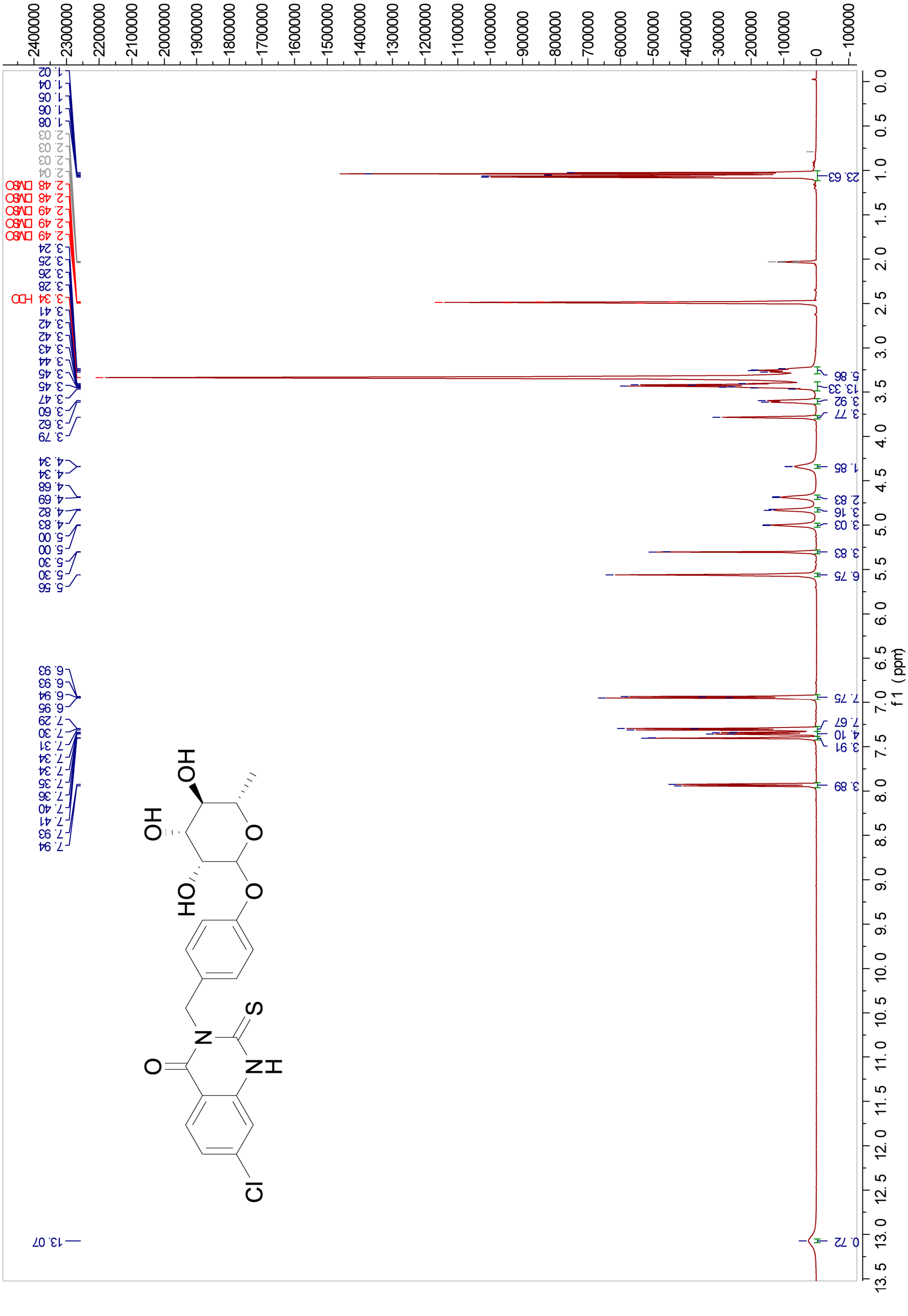

NMR result of MITC-05

^{1}H NMR (500 MHz, DMSO-d_6) δ 12.83 (d, J = 240.3 Hz, 1H), 7.93 (d, J = 8.5 Hz, 1H), 7.40 (d, J = 1.7 Hz, 1H), 7.35 (dd, J = 8.5, 1.8 Hz, 1H), 7.30 (d, J = 8.6 Hz, 2H), 6.94 (d, J = 8.6 Hz, 2H), 5.56 (s, 2H), 5.30 (s, 1H), 5.00 (s, 1H), 4.83 (s, 1H), 4.69 (s, 1H), 3.79 (s, 1H), 3.61 (1H), 3.43 (1H), 3.26 (1H), 1.07 – 1.04 (m, 3H)..; ^{13}C NMR (126 MHz, DMSO) δ 175.9, 158.8, 155.2, 140.0, 139.9, 129.7, 129.5, 128.9, 124.6, 116.1, 115.0, 114.4, 98.4, 71.8, 70.4, 70.2, 69.4, 48.1, 17.8.

^{13}C NMR of MITC-06

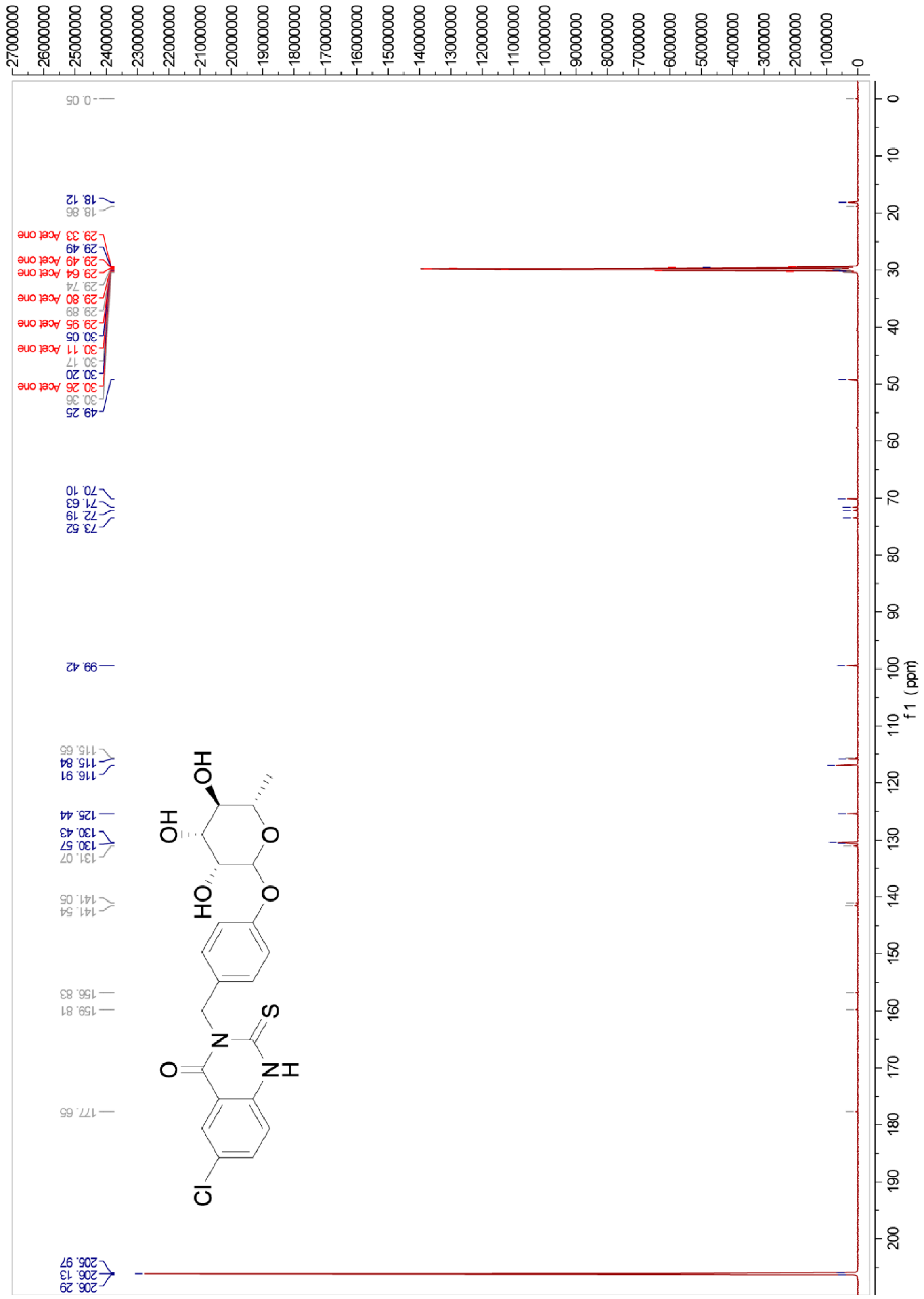

^{1}H NMR of MITC-06

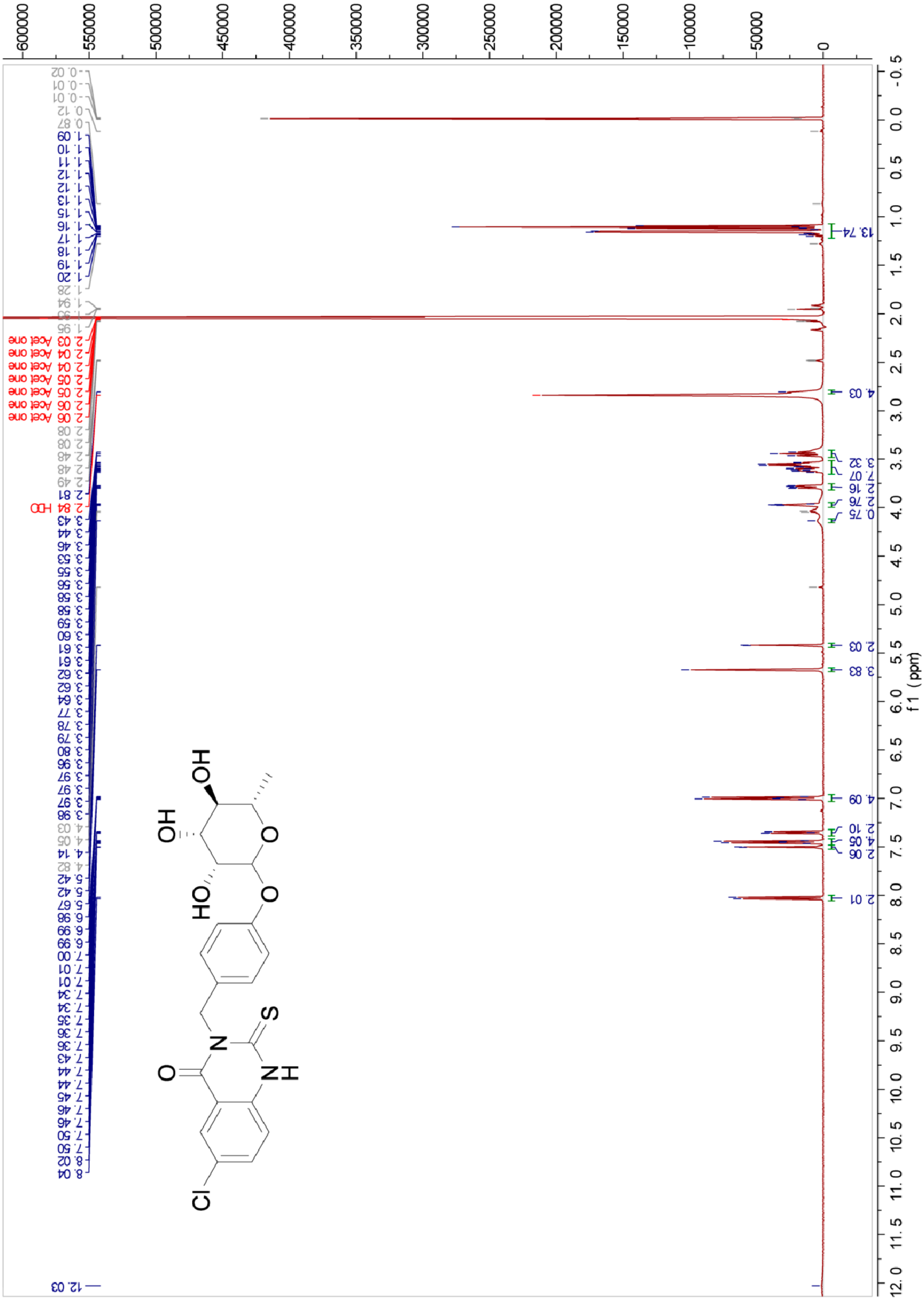

NMR result of MITC-06

^{1}H NMR (500 MHz, Acetone-d_6) δ 12.03 (s, 1H), 8.03 (d, J = 8.5 Hz, 1H), 7.50 (d, J = 1.9 Hz, 1H), 7.48 – 7.43 (m, 2H), 7.35 (dd, J = 8.5, 1.9 Hz, 1H), 7.04 – 6.95 (m, 2H), 5.67 (s, 2H), 5.42 (d, J = 1.7 Hz, 1H), 3.97 (1H), 3.79 (1H), 3.62 (1H), 3.56 (q, J = 7.0 Hz, 2H), 3.44 (1H), 1.15 (d, J = 6.2 Hz, 3H).;^{13}C NMR (126 MHz, Acetone) δ 177.6, 159.8, 156.8, 141.5, 141.0, 131.0, 130.5, 130.4, 125.4, 116.9, 115.8, 115.6, 99.4, 73.5, 72.1, 71.6, 70.1, 49.2, 18.1.

^{13}C NMR of MITC-07

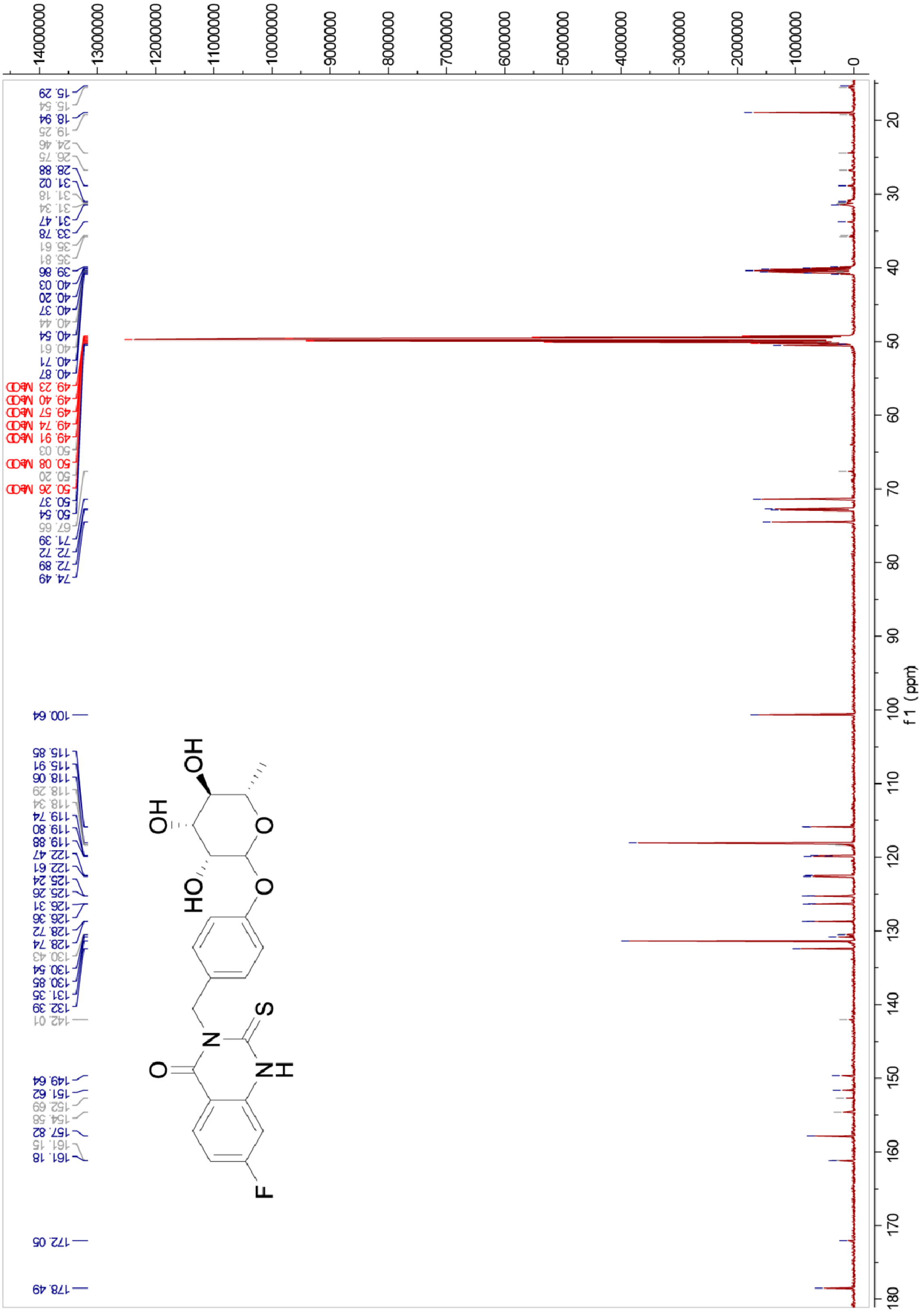

^{1}H NMR of MITC-07

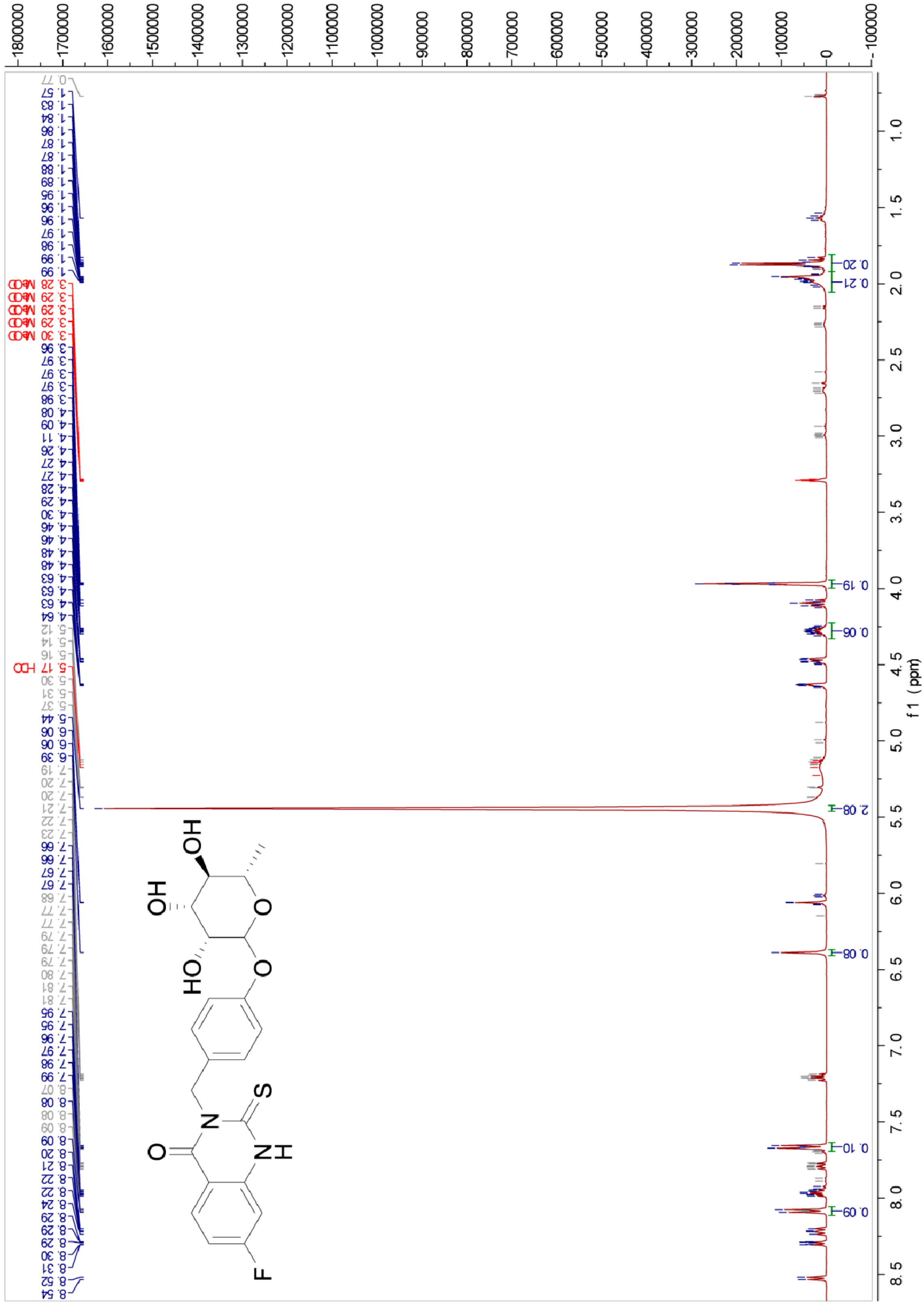

NMR result of MITC-07

^{1}H NMR (500 MHz, Mode) δ 7.86 (dt, J = 8.1, 1.0 Hz, 1H), 7.53 (ddd, J = 10.7, 8.1, 1.3 Hz, 1H), 7.45 – 7.39 (m, 2H), 7.28 (td, J = 8.1, 4.7 Hz, 1H), 7.03 – 6.93 (m, 2H), 5.72 (s, 2H), 5.38 (d, J = 1.9 Hz, 1H), 3.96 (dd, J = 3.5, 1.8 Hz, 1H), 3.81 (1H), 3.66 – 3.56 (m, 1H), 3.46 – 3.38 (m, 1H), 1.22 – 1.18 (m, 3H).;^{13}C NMR (126 MHz, MeOD) δ 178.4, 157.8, 132.3, 131.3, 128.7, 126.3, 122.6, 122.4, 119.8, 119.7, 118.0, 115.8, 100.6, 74.4, 72.8, 72.7, 71.3, 49.2, 18.9.

^{13}C NMR of MITC-08

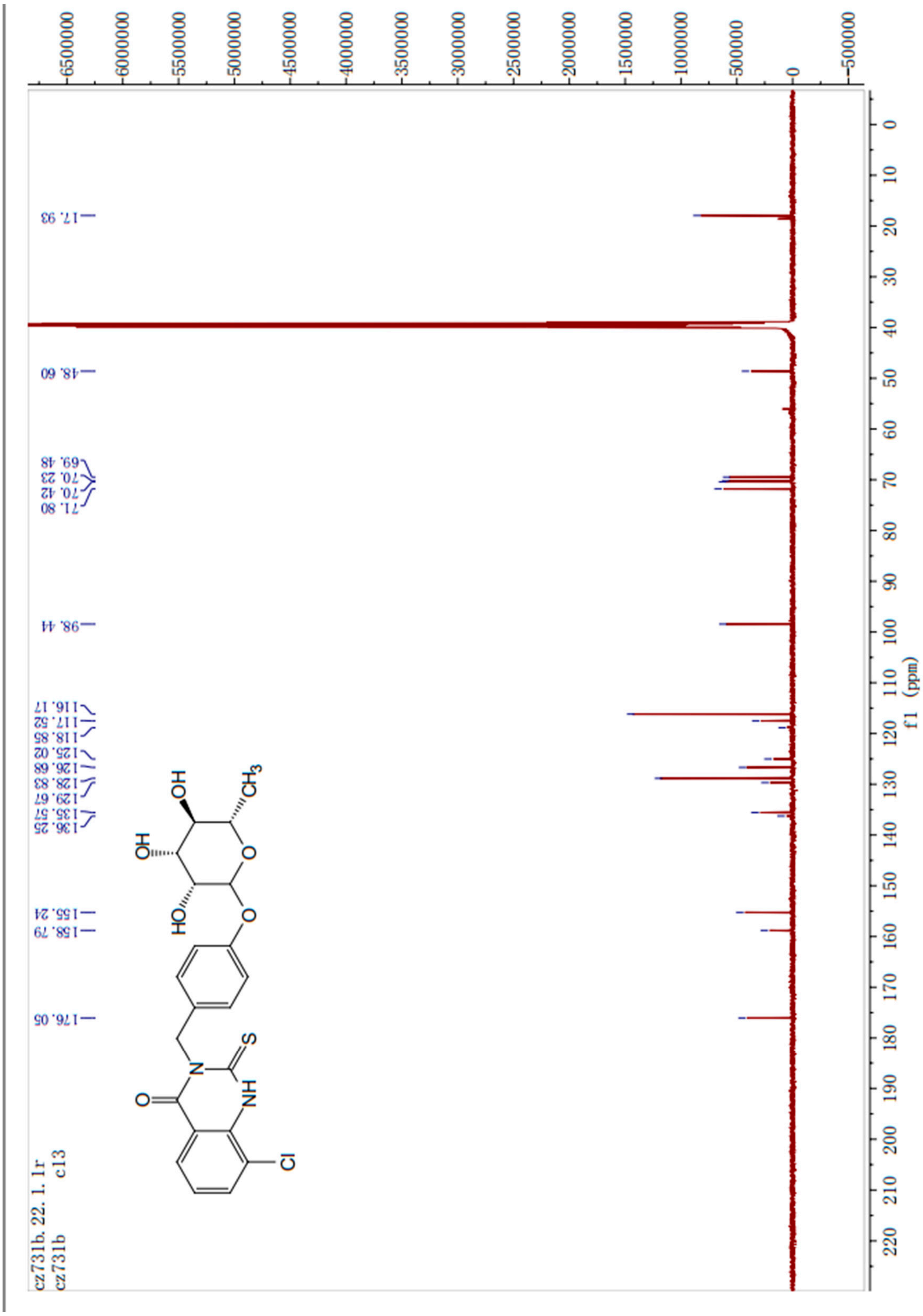

^{1}H NMR of MITC-08

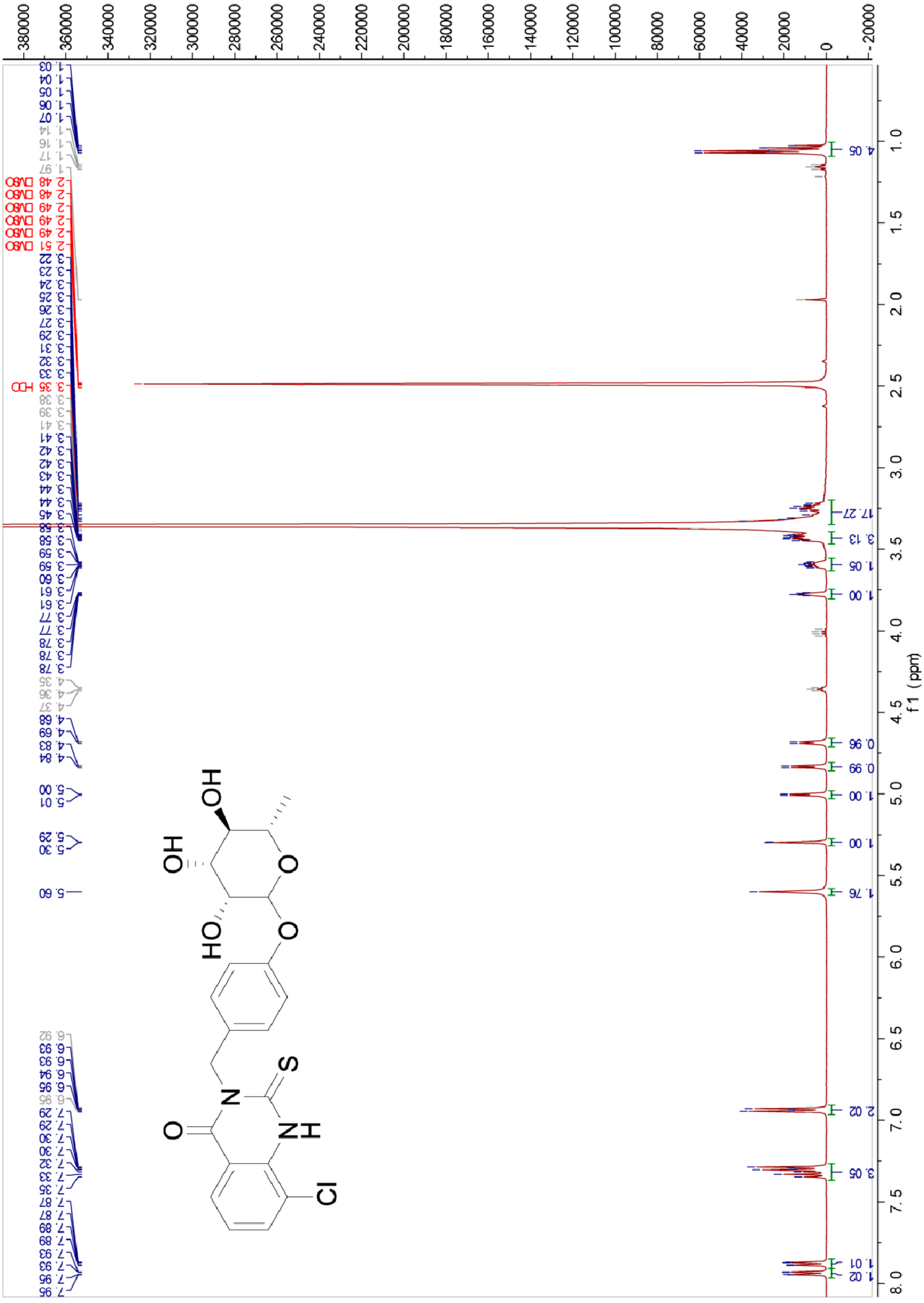

NMR result of MITC-08

^{1}H NMR (500 MHz, DMSO-d_6) δ 11.66 (s, 1H), 7.93 (dd, J = 7.9, 1.4 Hz, 1H), 7.87 (dd, J = 7.9, 1.4 Hz, 1H), 7.33 (d, J = 7.9 Hz, 1H), 7.30 (dd, J = 8.8, 2.2 Hz, 2H), 6.98 – 6.90 (m, 2H), 5.60 (s, 2H), 5.30 (d, J = 1.7 Hz, 1H), 5.01 (d, J = 4.4 Hz, 1H), 4.83 (d, J = 5.8 Hz, 1H), 4.69 (d, J = 6.0 Hz, 1H), 3.78 (td, J = 3.8, 3.4, 1.8 Hz, 1H), 3.59 (1H), 3.48 – 3.40 (m, 1H), 1.08 – 1.05 (m, 3H).;^{13}C NMR (126 MHz, DMSO) δ 176.0, 158.7, 155.2, 136.2, 135.5, 129.6, 128.8, 126.6, 125.0, 118.8, 117.5, 116.1, 98.4, 71.8, 70.4, 70.2, 69.4, 48.6, 17.9

^{13}C NMR of MITC-09

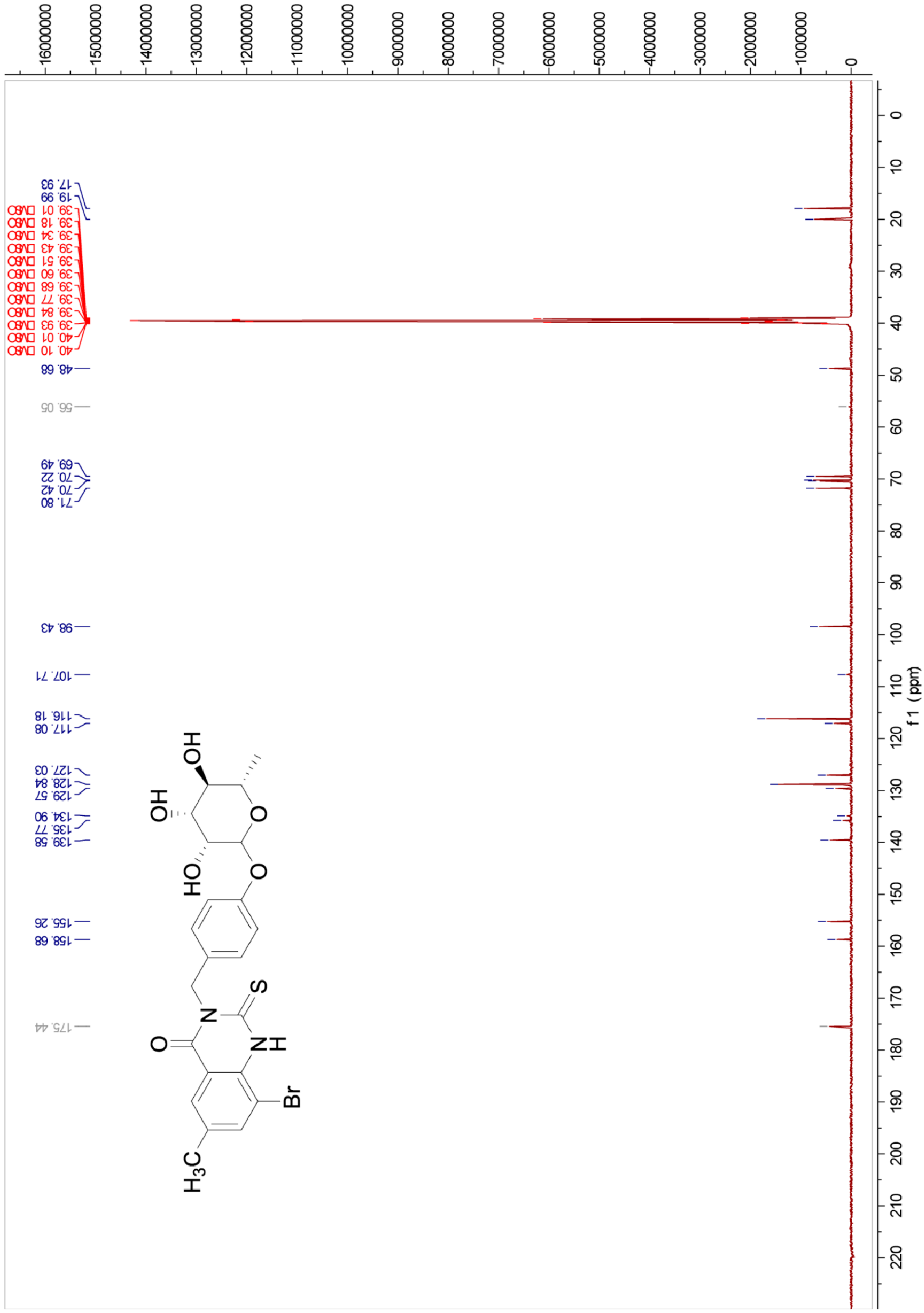

^{1}H NMR of MITC-09

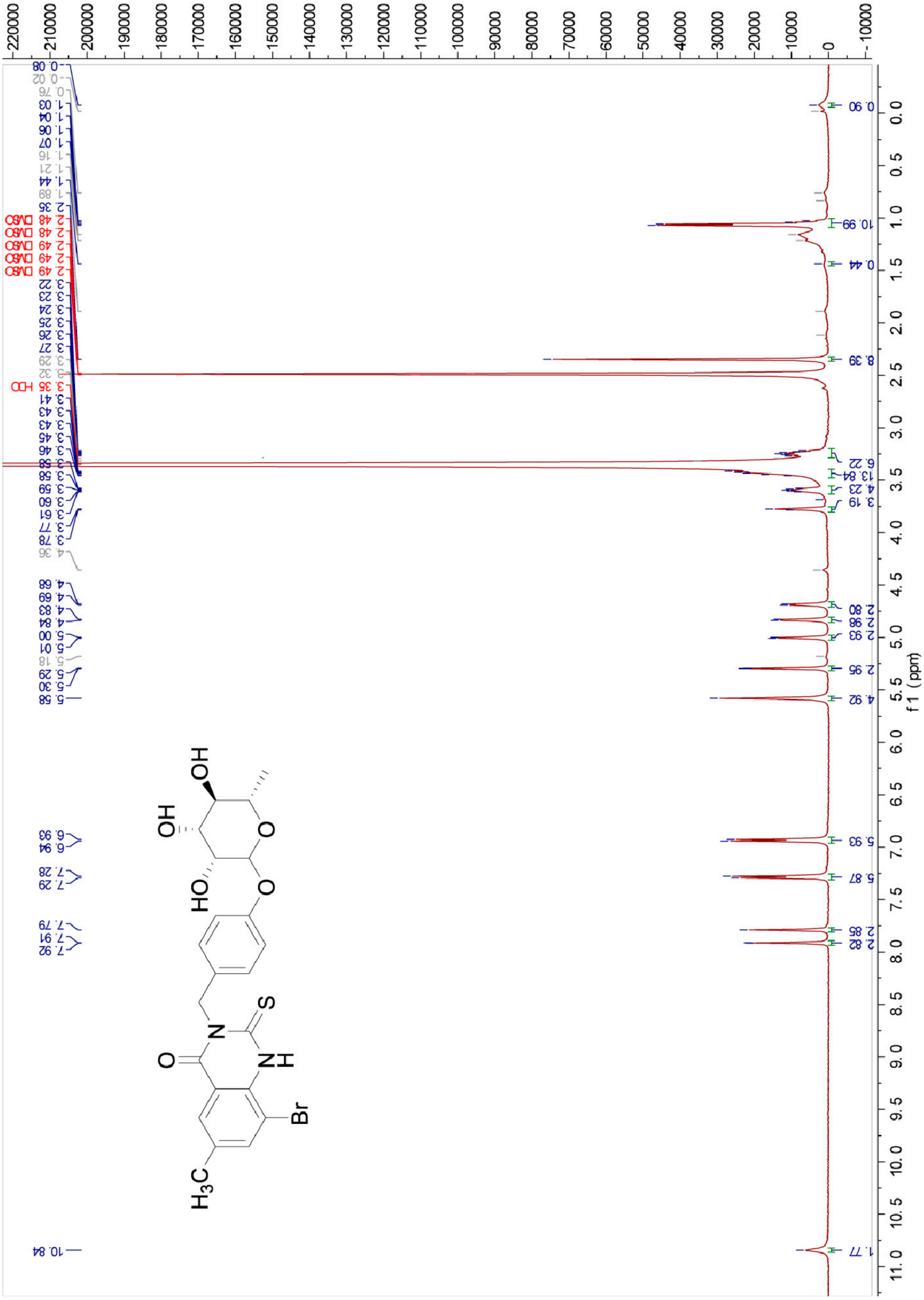

NMR result of MITC-09

^{1}H NMR (500 MHz, DMSO-d_6) δ 10.84 (s, 1H), 7.95 – 7.89 (m, 1H), 7.79 (s, 1H), 7.28 (d, J = 8.3 Hz, 2H), 6.93 (d, J = 8.3 Hz, 2H), 5.58 (s, 2H), 5.33 – 5.27 (m, 1H), 5.00 (d, J = 4.4 Hz, 1H), 4.83 (d, J = 5.9 Hz, 1H), 4.69 (d, J = 6.0 Hz, 1H), 3.78 (d, J = 4.6 Hz, 1H), 3.59 (1H), 3.43 (1H), 2.35 (s, 3H), 1.06 (d, J = 6.3 Hz, 3H).;^{13}C NMR (126 MHz, DMSO) δ 175.4, 158.6, 155.2, 139.5, 135.7, 134.9, 129.5, 128.8, 127.0, 117.0, 116.1, 107.7, 98.4, 71.8, 70.4, 70.2, 69.4, 48.6, 19.9, 17.9.

^{13}C NMR of MITC-10

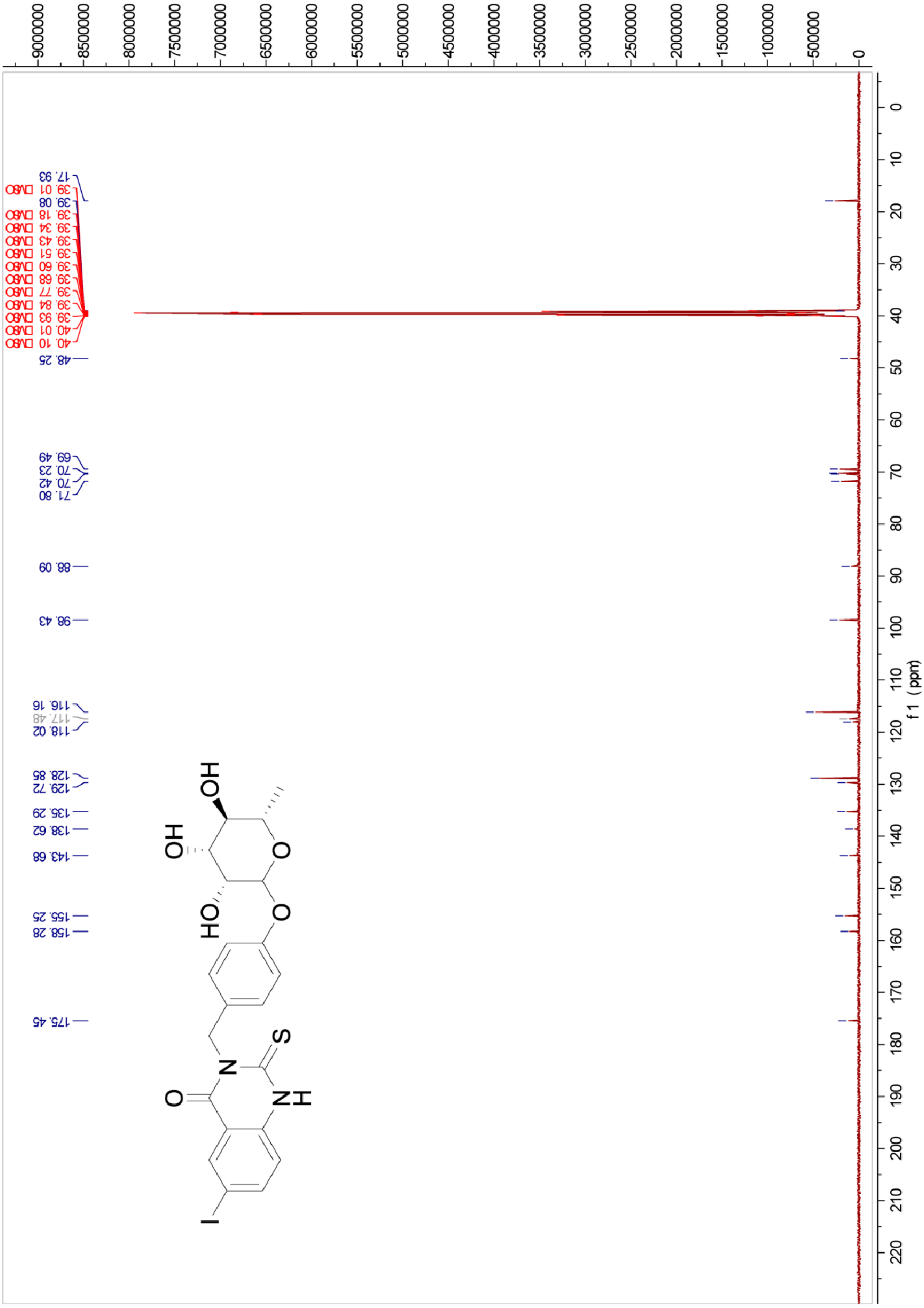

^{1}H NMR of MITC-10

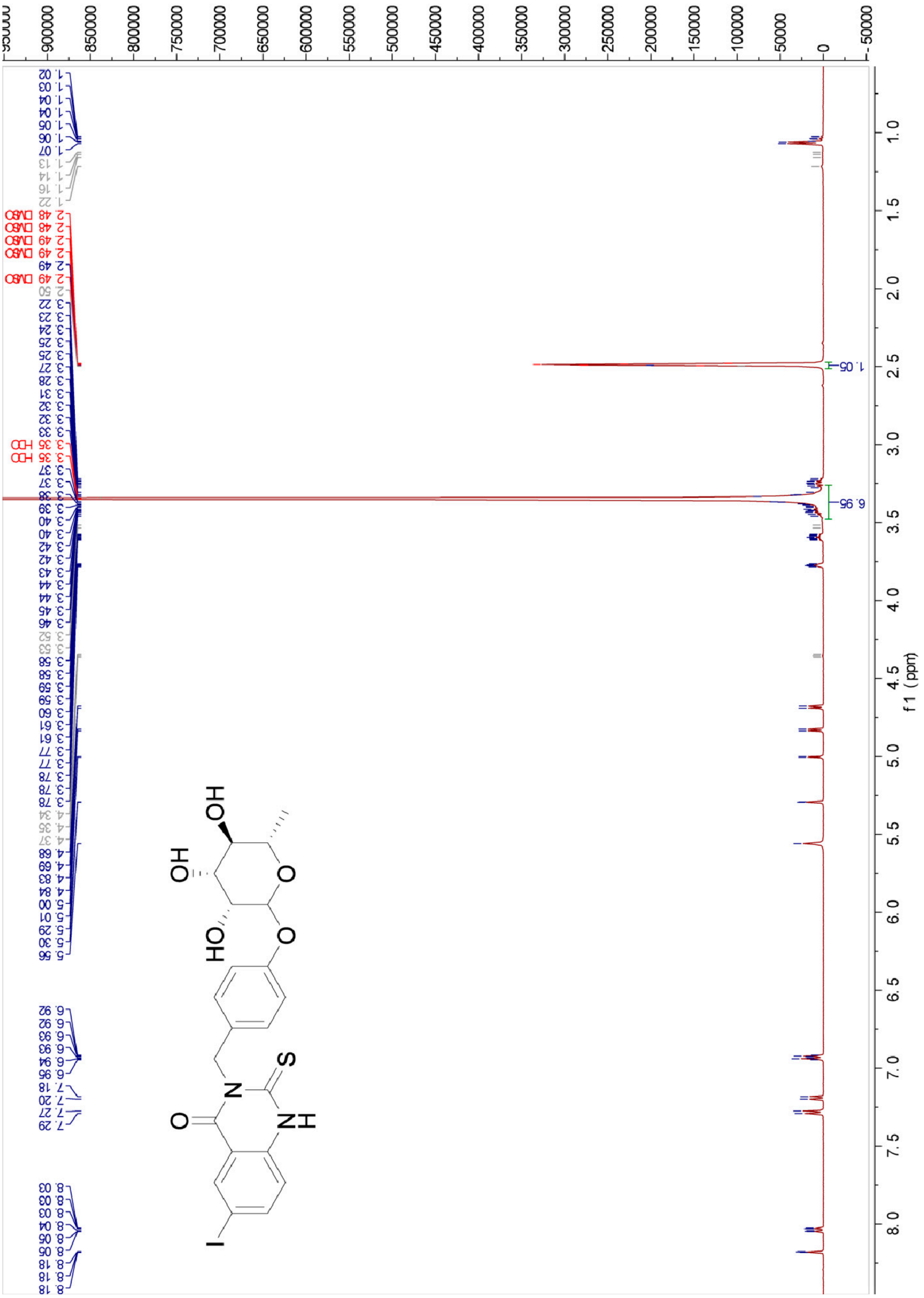

NMR result of MITC-10

^{1}H NMR (500 MHz, DMSO-d_6) δ 13.09 (s, 1H), 8.18 (d, J = 2.0 Hz, 1H), 8.04 (dd, J = 8.6, 2.0 Hz, 1H), 7.31 – 7.26 (m, 2H), 7.19 (d, J = 8.6 Hz, 1H), 6.96 – 6.91 (m, 2H), 5.56 (s, 2H), 5.29 (d, J = 1.8 Hz, 1H), 5.00 (d, J = 4.4 Hz, 1H), 4.83 (d, J = 5.8 Hz, 1H), 4.68 (d, J = 6.0 Hz, 1H), 3.77 (1H), 3.59 (1H), 3.46 – 3.41 (m, 1H), 3.24 (1H), 1.07 (d, J = 6.1 Hz, 3H).;^{13}C NMR (126 MHz, DMSO) δ 175.4, 158.2, 155.2, 143.6, 138.6, 135.2, 132.8, 129.7, 128.8, 118.0, 117.4, 116.1, 98.4, 71.8. 70.4, 70.2, 69.4, 48.2, 17.9.

^{13}C NMR of MITC-11

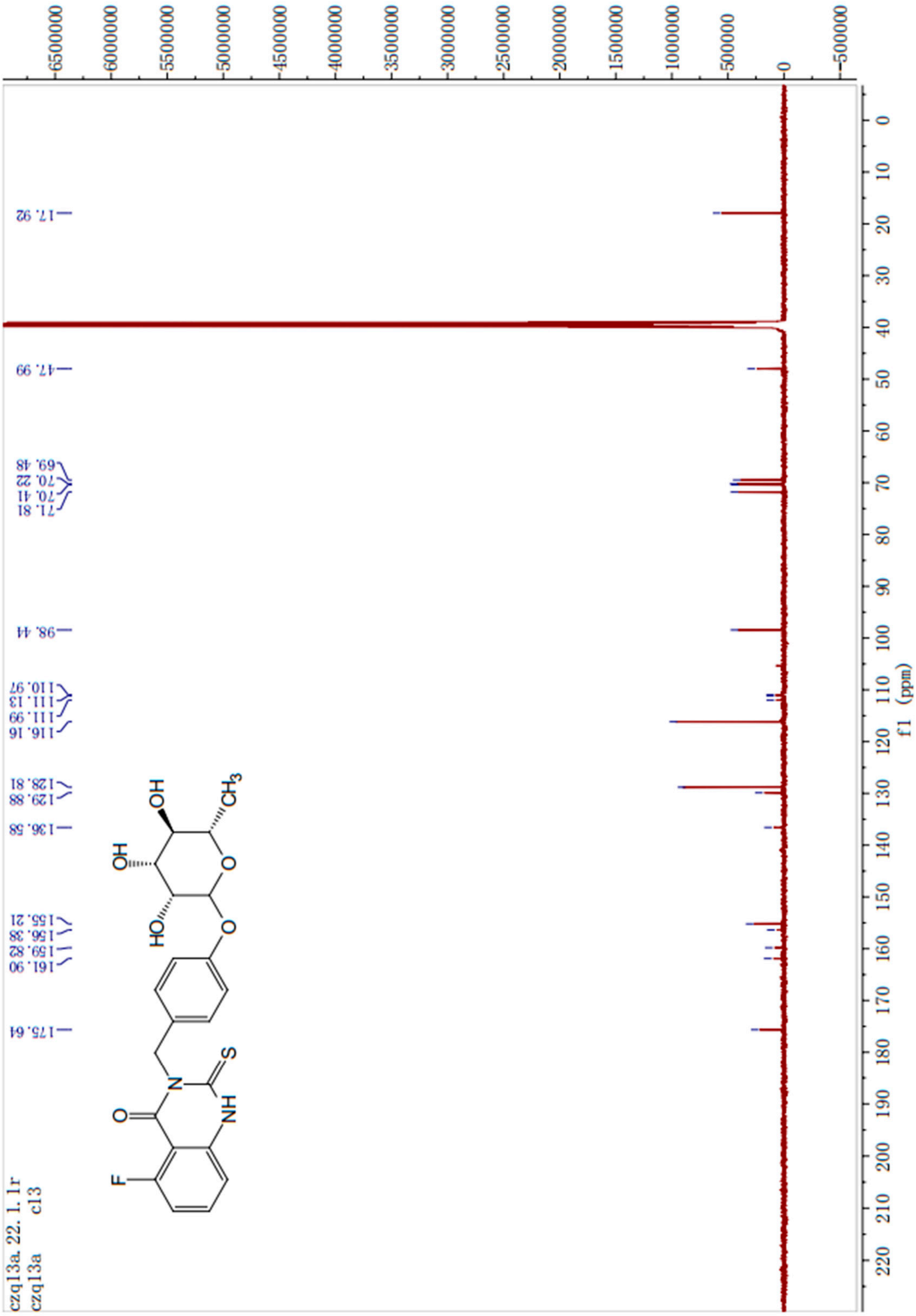

^{1}H NMR of MITC-11

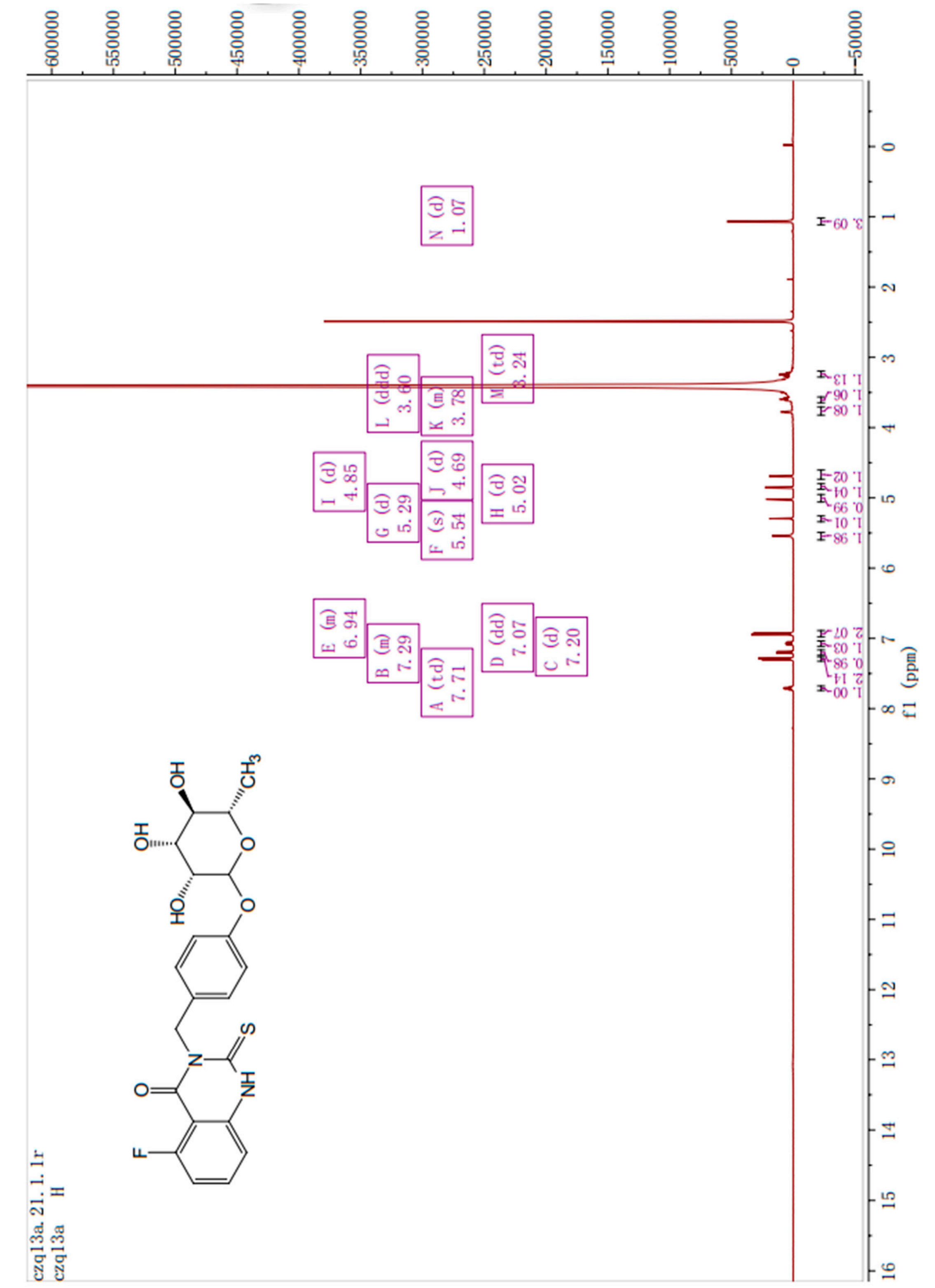

NMR result of MITC-11

^{1}H NMR (500 MHz, DMSO-d_6) δ 7.71 (td, J = 8.3, 5.2 Hz, 1H), 7.32 – 7.26 (m, 2H), 7.20 (d, J = 8.3 Hz, 1H), 7.07 (dd, J = 11.1, 8.2 Hz, 1H), 6.98 – 6.89 (m, 2H), 5.54 (s, 2H), 5.29 (d, J = 1.7 Hz, 1H), 5.02 (d, J = 4.4 Hz, 1H), 4.85 (d, J = 5.8 Hz, 1H), 4.69 (d, J = 6.1 Hz, 1H), 3.82 – 3.71 (m, 1H), 3.60 (1H), 3.24 (1H), 1.07 (d, J = 6.1 Hz, 3H)..;^{13}C NMR (126 MHz, DMSO) δ 175.6, 161.9, 159.8, 156.3, 155.2, 136.5, 129.8, 128.8, 116.1, 111.9, 111.1, 110.9, 98.4, 71.8, 70.4, 70.2, 69.4, 47.9, 17.9.

^{13}C NMR of MITC-12

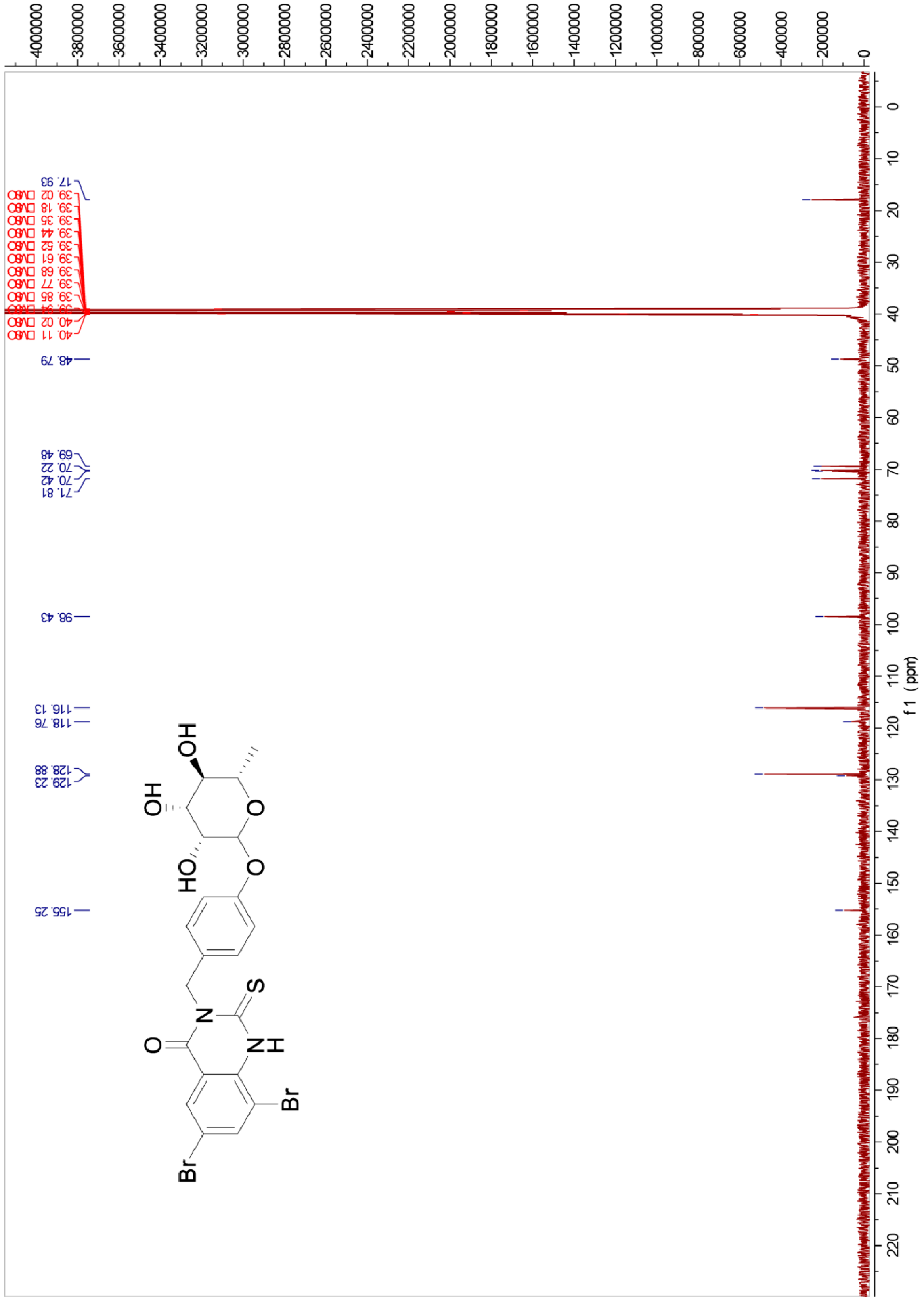

^{1}H NMR of MITC-12

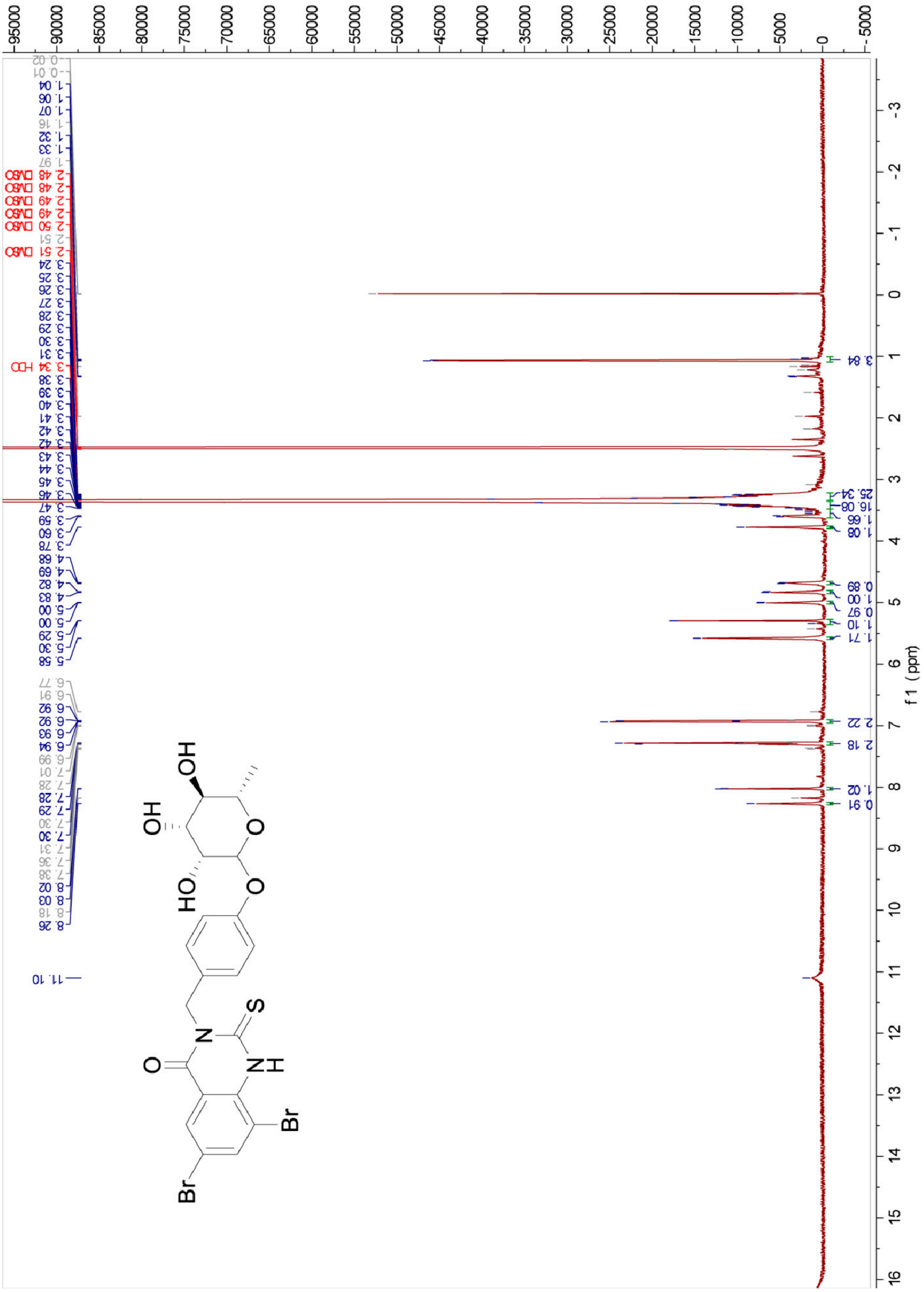

NMR result of MITC-12

^{1}H NMR (500 MHz, DMSO-d_6) δ 11.10 (s, 1H), 8.26 (s, 1H), 8.02 (d, J = 2.1 Hz, 1H), 7.34 – 7.24 (m, 2H), 6.98 – 6.88 (m, 2H), 5.58 (s, 2H), 5.30 (d, J = 1.7 Hz, 1H), 5.00 (d, J = 4.3 Hz, 1H), 4.83 (d, J = 5.9 Hz, 1H), 4.68 (d, J = 5.9 Hz, 1H), 3.78 (s, 1H), 3.59 (1H), 3.48 – 3.41 (m, 1H), 1.06 (d, J = 6.2 Hz, 3H)..;^{13}C NMR (126 MHz, DMSO) δ 175.9, 172.8, 168.0, 155.2, 149.3, 142.4, 140.2, 134.1, 129.2, 128.8, 118.7, 116.1, 98.4, 71.8, 70.4, 70.2, 69.4, 48.7, 17.9.

^{13}C NMR of MITC-13

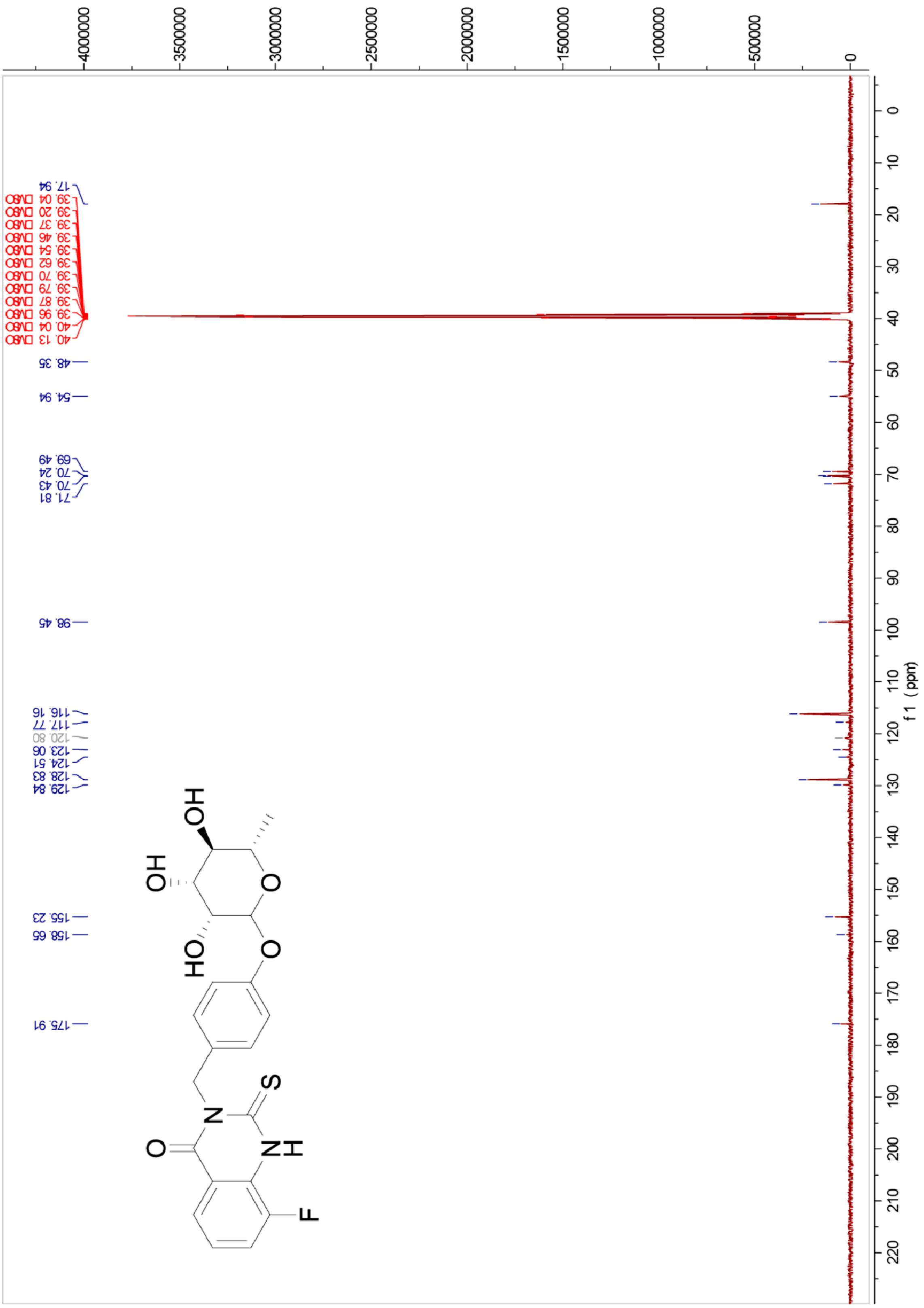

^{1}H NMR of MITC-13

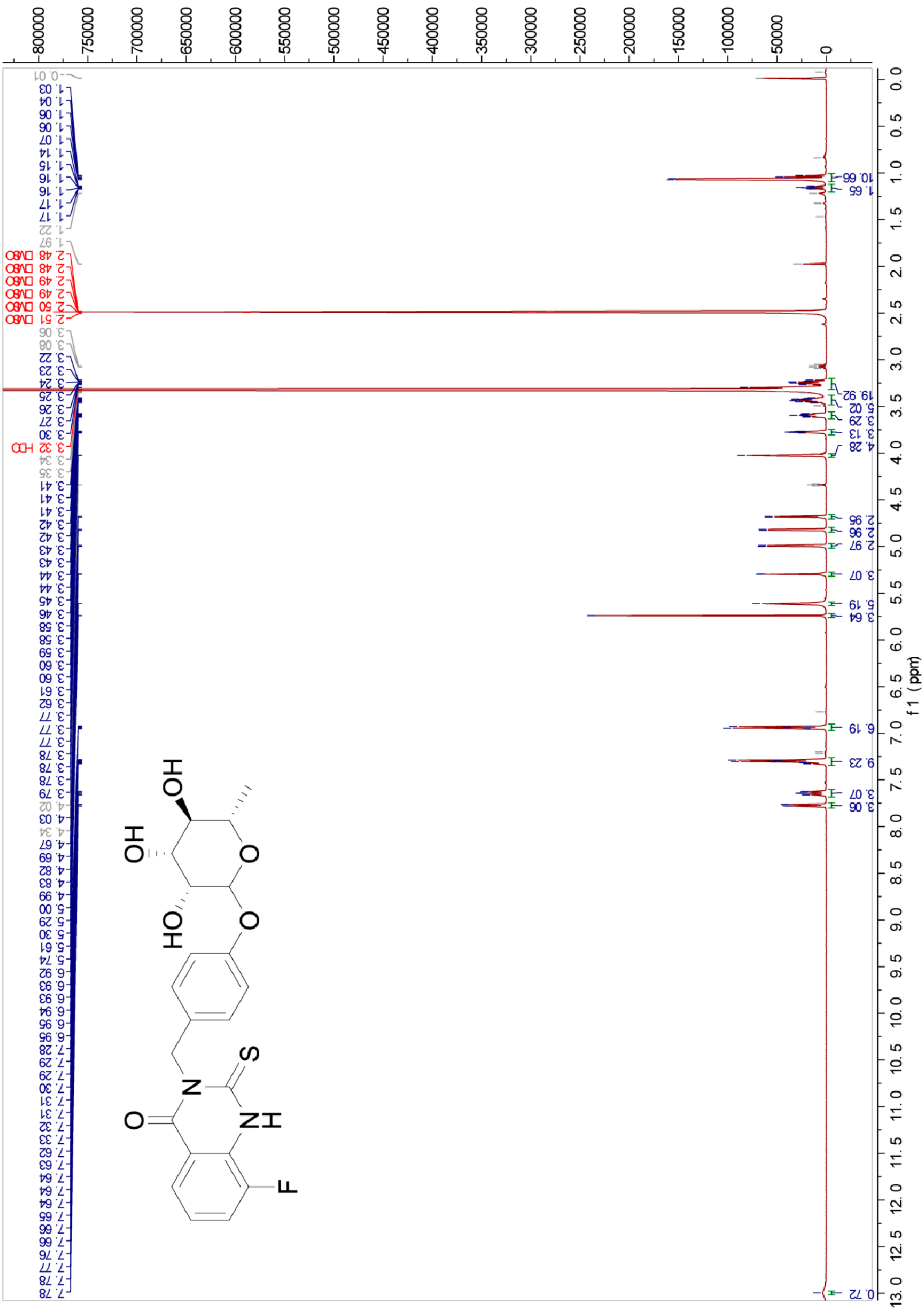

NMR result of MITC-13

^{1}H NMR (500 MHz, DMSO-d_6) δ 12.99 (s, 1H), 7.77 (dd, J = 8.0, 1.3 Hz, 1H), 7.64 (ddd, J = 10.6, 8.0，1.3 Hz, 1H), 7.30 (q, J = 4.1, 3.2 Hz, 2H), 7.29 (s, 1H), 6.97 – 6.89 (m, 2H), 5.61 (s, 2H), 5.30 (d, J = 1.8 Hz, 1H), 4.99 (d, J = 4.4 Hz, 1H), 4.82 (d, J = 5.7 Hz, 1H), 4.68 (d, J = 6.0 Hz, 1H), 4.02 (d, J = 1.9 Hz, 1H), 3.78 (1H), 3.60 (1H), 3.48 – 3.39 (m, 1H), 1.06 (m, J = 9.7, 6.6 Hz, 3H).;^{13}C NMR (126 MHz, DMSO) δ 175.9, 163.2, 155.2, 134.8, 129.8, 128.8, 124.4, 123.0, 120.8, 120.2, 117.7, 116.1, 98.4, 71.8, 70.4, 70.2, 69.4, 48.3, 17.9.

^{13}C NMR of MITC-14

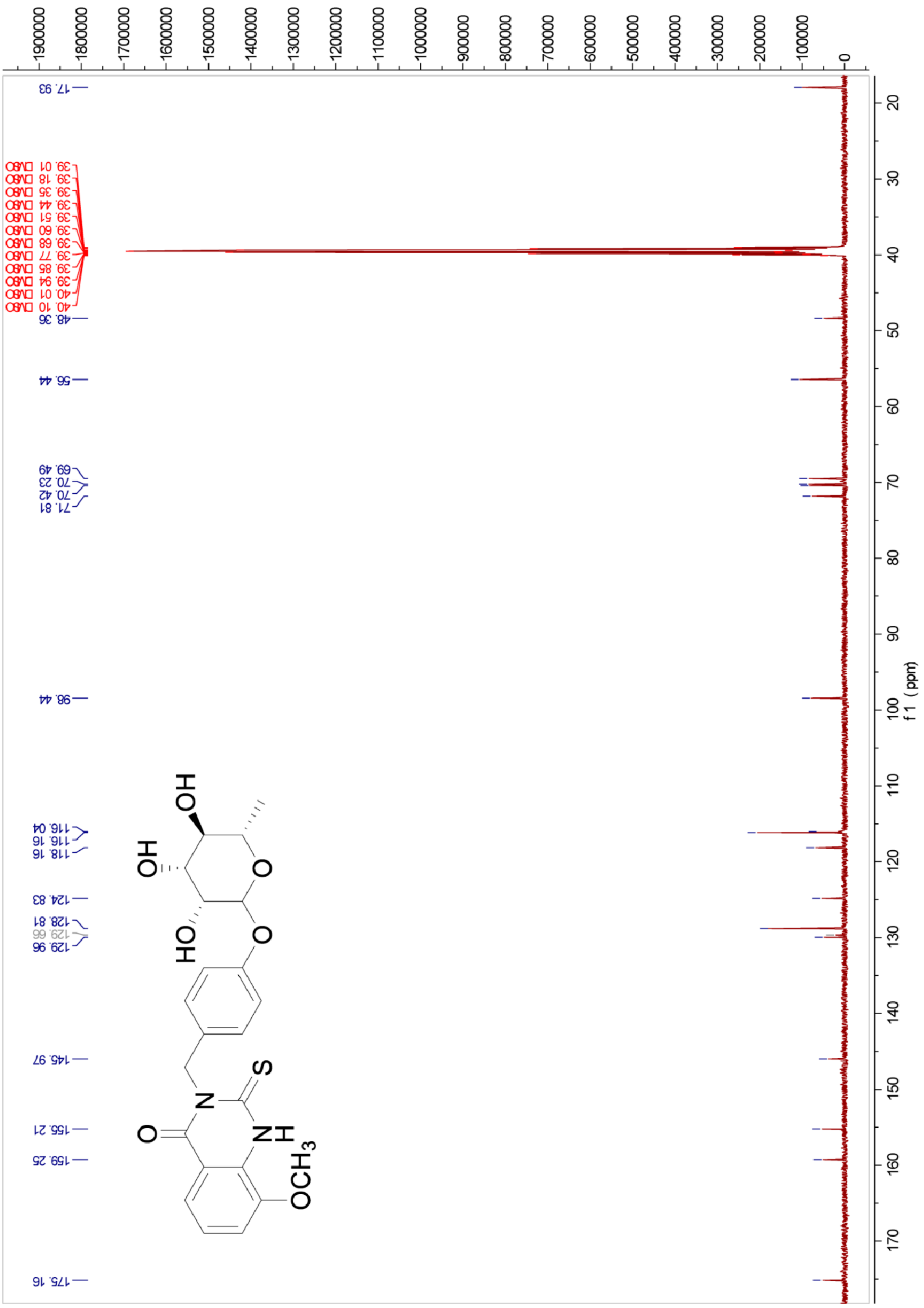

^{1}H NMR of MITC-14

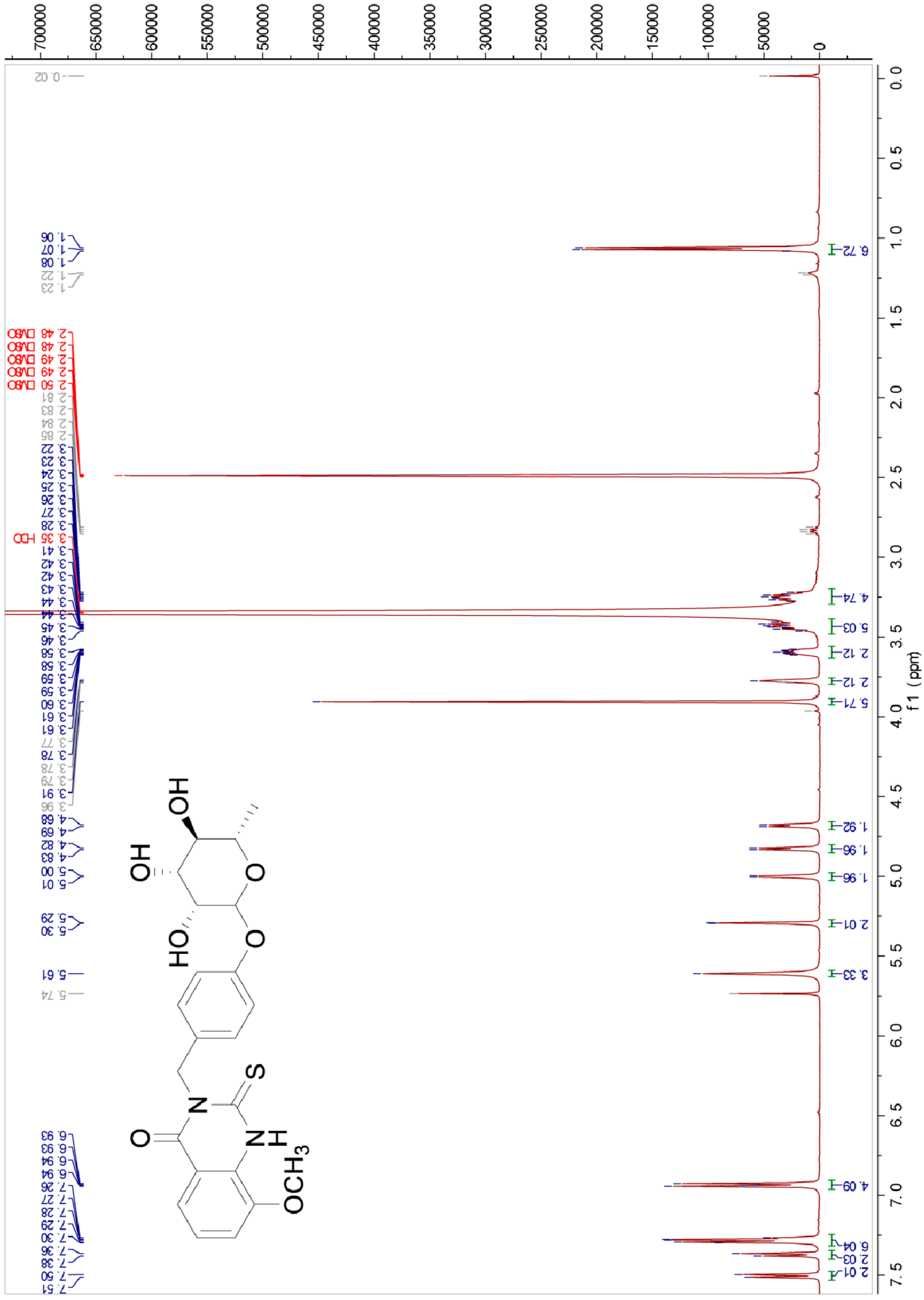

NMR result of MITC-14

^{1}H NMR (500 MHz, DMSO-d_6) δ 11.67 (s, 1H), 7.50 (d, J = 8.0 Hz, 1H), 7.37 (d, J = 8.0 Hz, 1H), 7.30 – 7.28 (m, 2H), 7.27 (s, 1H), 6.97 – 6.90 (m, 2H), 5.61 (s, 2H), 5.29 (d, J = 1.8 Hz, 1H), 5.00 (d, J = 4.4 Hz, 1H), 4.83 (d, J = 5.8 Hz, 1H), 4.68 (d, J = 6.0 Hz, 1H), 3.91 (s, 3H), 3.78 (1H), 3.59 (1H), 3.46 – 3.41 (m, 1H), 3.24 (1H), 1.07 (d, J = 6.2 Hz, 3H).;^{13}C NMR (126 MHz, DMSO) δ 175.1, 159.2, 155.21, 149.3, 145.9, 129.9, 129.6, 128.8, 124.8, 118.1, 116.1, 116.0, 98.4, 71.8, 70.4, 70.2, 69.4, 56.4, 48.3, 17.9.

^{13}C NMR of MITC-15

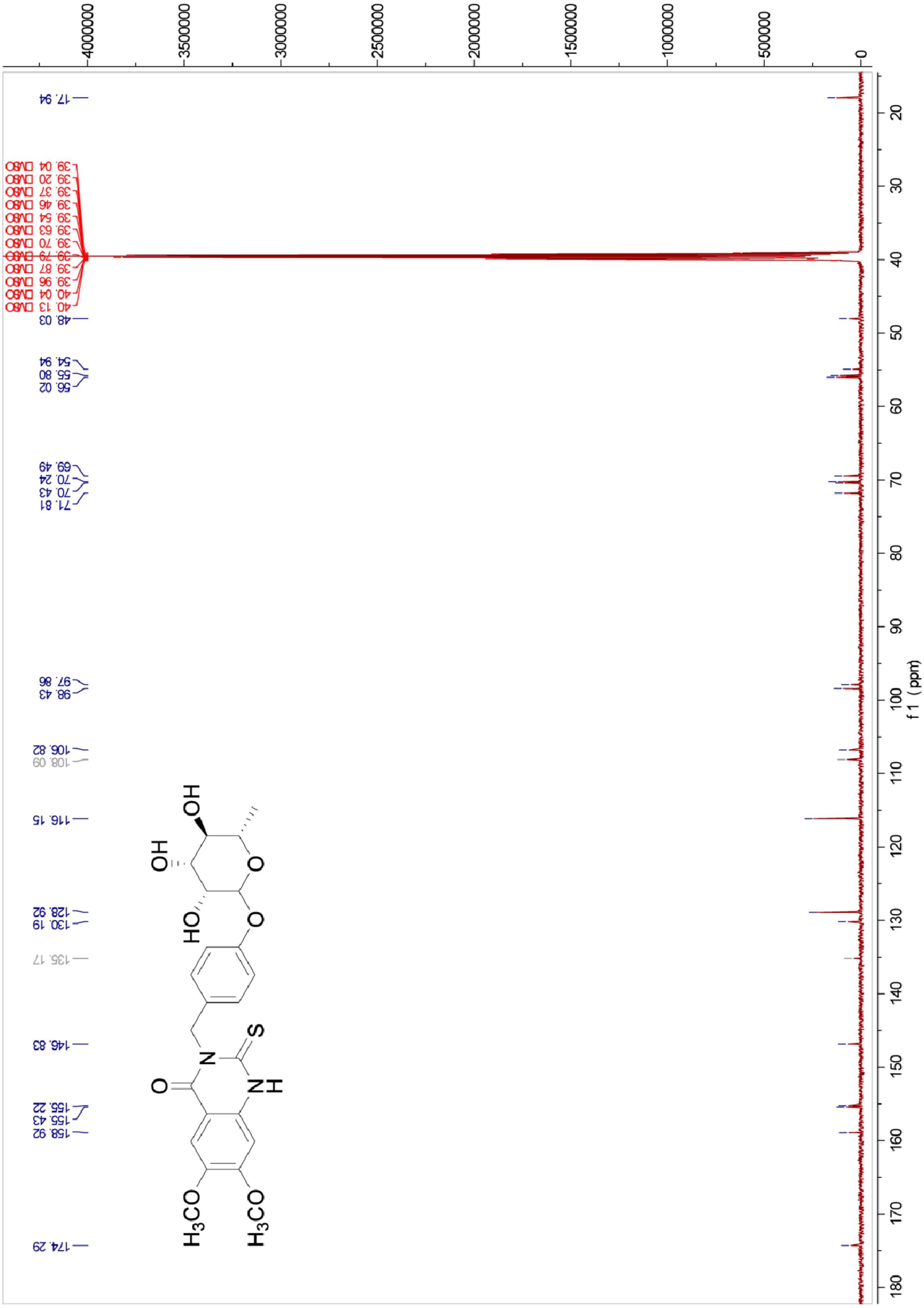

^{1}H NMR of MITC-15

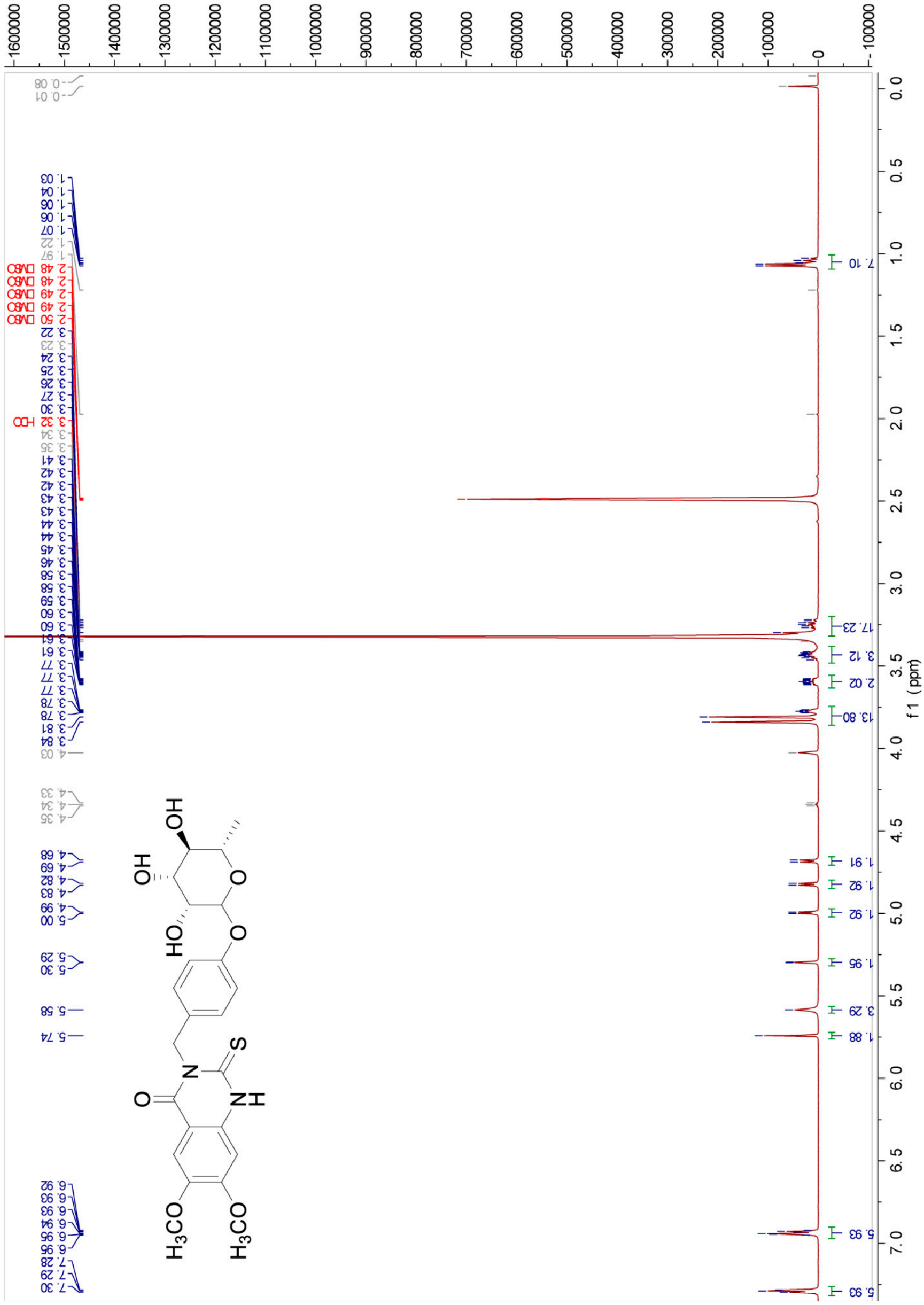

NMR result of MITC-15

^{1}H NMR (500 MHz, DMSO-d_6) δ 12.85 (s, 1H), 7.30 (s, 1H), 7.29 (d, J = 3.2 Hz, 2H), 6.94 (d, J = 2.3 Hz, 2H), 6.93 (d, J = 3.2 Hz, 1H), 5.58 (s, 2H), 5.30 (d, J = 1.8 Hz, 1H), 5.00 (d, J = 4.4 Hz, 1H), 4.82 (d, J = 5.8 Hz, 1H), 4.68 (d, J = 6.0 Hz, 1H), 4.03 (s, 1H), 3.84 (s, 3H), 3.81 (s, 3H), 3.60 (1H), 3.47 – 3.40 (m, 1H), 3.30 (s, 1H), 1.07 (d, J = 6.2 Hz, 3H).;^{13}C NMR (126 MHz, DMSO) δ 174.2, 158.9, 155.4, 155.2, 146.8, 135.1, 130.1, 128.9, 116.1, 112.7, 108.0, 106.8, 98.4, 71.8, 70.4, 70.2, 69.4, 48.0, 17.9.

^{13}C NMR of MITC-16

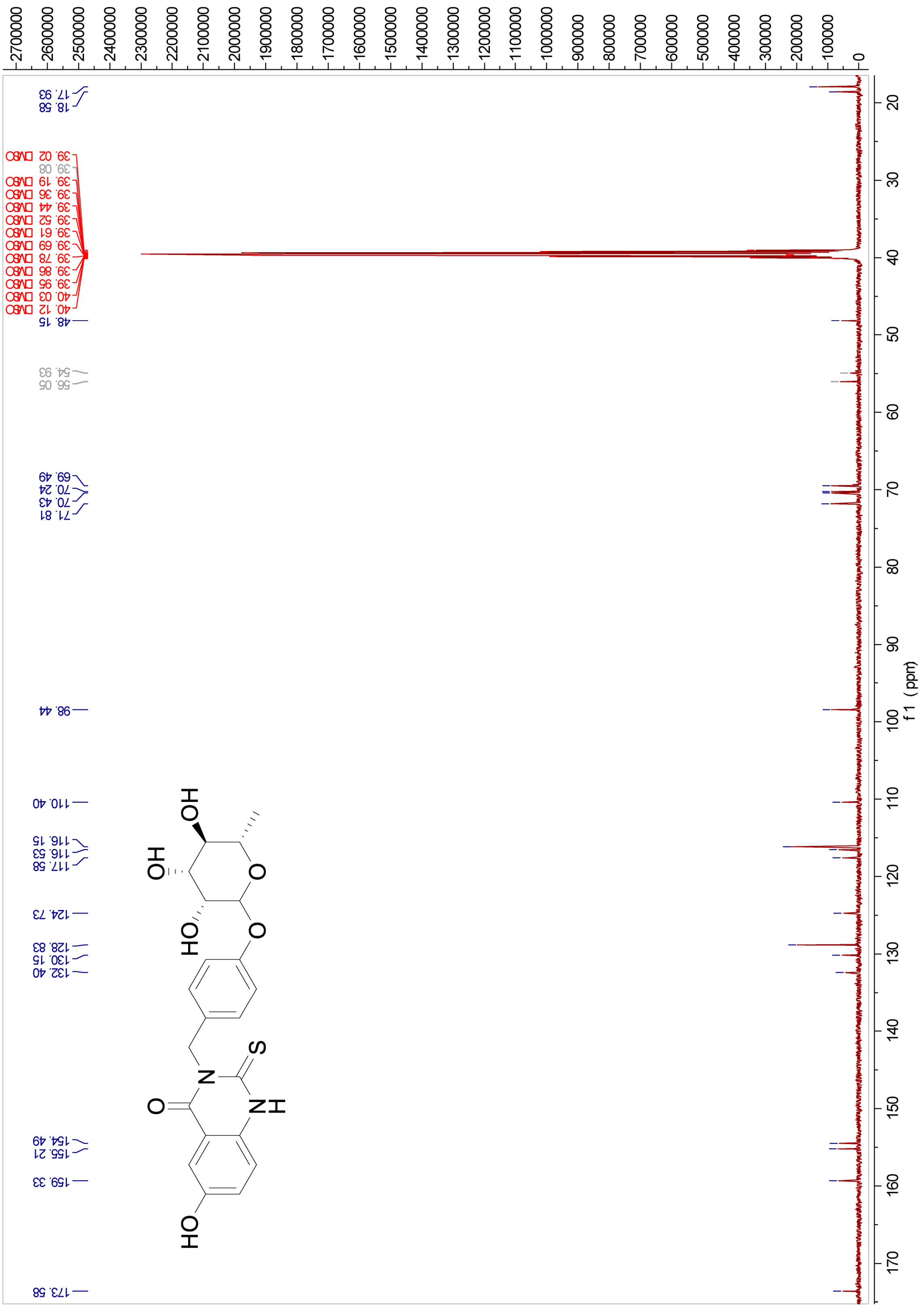

^{1}H NMR of MITC-16

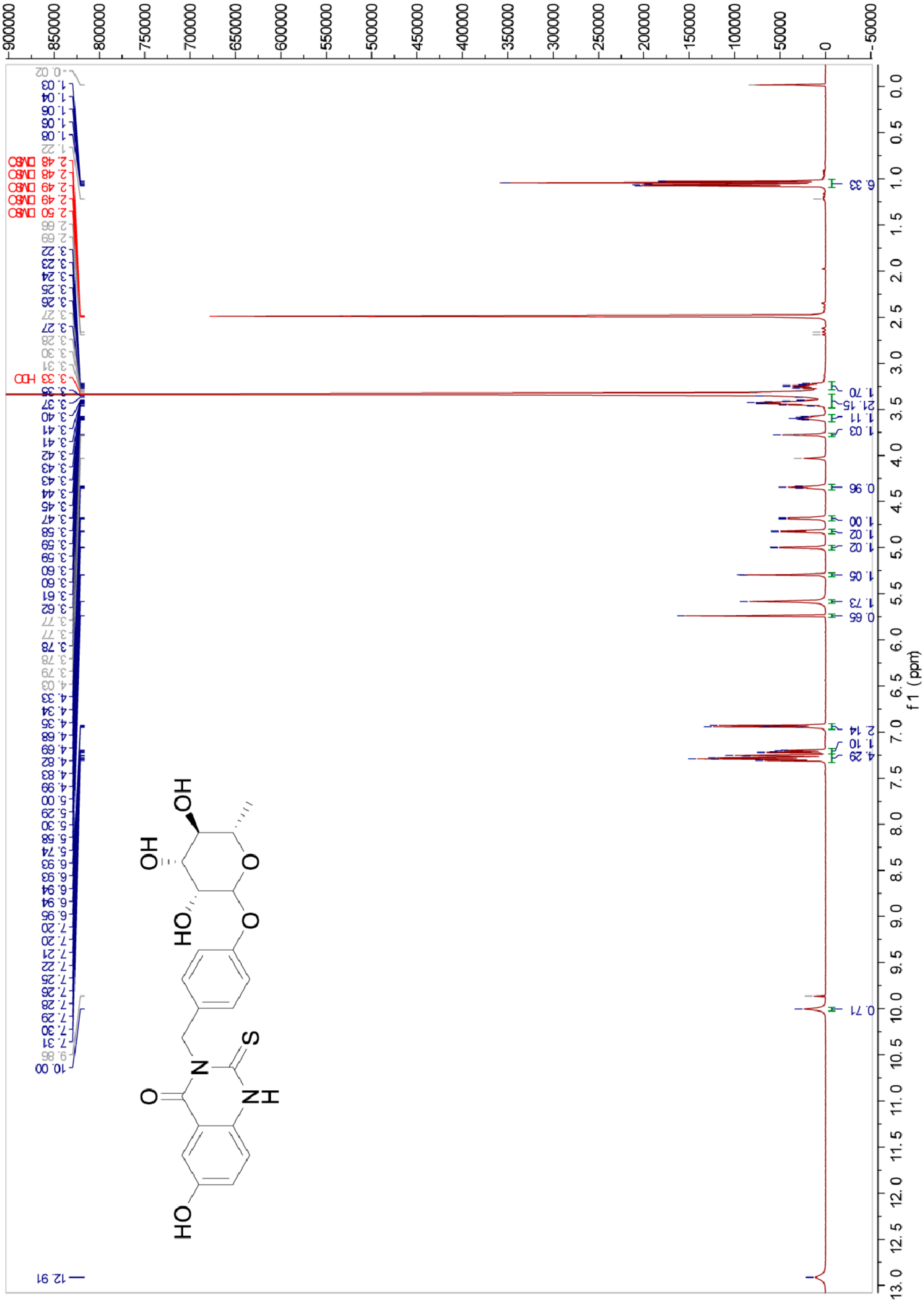

NMR result of MITC-16

^{1}H NMR (500 MHz, DMSO-d_6) δ 10.00 (s, 1H), 7.31 – 7.29 (m, 2H), 7.28 (d, J = 2.0 Hz, 1H), 7.25 (d, J = 2.0 Hz, 1H), 7.21 (dd, J = 8.8, 2.7 Hz, 1H), 6.95 – 6.92 (m, 2H), 5.58 (s, 2H), 5.29 (d, J = 2.0 Hz, 1H), 5.00 (d, J = 4.4 Hz, 1H), 4.82 (d, J = 5.8 Hz, 1H), 4.68 (d, J = 6.0 Hz, 1H), 4.34 (t, J = 5.1 Hz, 1H), 3.80 – 3.76 (m, 1H), 3.60 (1H), 3.24 (1H), 1.08 – 1.05 (m, 3H)..;^{13}C NMR (126 MHz, DMSO) δ 173.5, 159.3, 155.2, 154.4, 132.4, 130.1, 128.8, 124.7, 117.5, 116.5, 116.1, 110.4, 98.4, 71.8, 70.4, 70.2, 69.4, 48.1, 17.9.
